# Identification of oxidative phosphorylation-related genes in moyamoya disease by combining bulk RNA-sequencing analysis and machine learning

**DOI:** 10.3389/fgene.2024.1417329

**Published:** 2024-06-10

**Authors:** Zhiguang Han, Junze Zhang, Yutao Su, Zhenyu Zhou, Yanru Wang, Shaoqi Xu, Yuanli Zhao, Shihao He, Rong Wang

**Affiliations:** ^1^ Department of Neurosurgery, Beijing Tiantan Hospital, Capital Medical University, Beijing, China; ^2^ Department of Neurosurgery, The 82nd Group Army Hospital, Baoding, China; ^3^ Suzhou Vocational Health College, Suzhou, China; ^4^ Department of Neurosurgery, Peking Union Medical College Hospital, Peking Union Medical College and Chinese Academy of Medical Sciences, Beijing, China

**Keywords:** moyamoya disease, oxidative phosphorylation, pathogenesis, RNA sequencing, immunity

## Abstract

**Introduction:** Moyamoya disease (MMD) is a chronic cerebrovascular disease that can lead to ischemia and hemorrhagic stroke. The relationship between oxidative phosphorylation (OXPHOS) and MMD pathogenesis remains unknown.

**Methods:** The gene expression data of 60 participants were acquired from three Gene Expression Omnibus (GEO) datasets, including 36 and 24 in the MMD and control groups. Differentially expressed genes (DEGs) between MMD patients MMD and control groups were identified. Machine learning was used to select the key OXPHOS-related genes associated with MMD from the intersection of DEGs and OXPHOS-related gene sets. Gene ontology (GO), Kyoto encyclopedia of genes and genomes (KEGG), gene set enrichment analysis (GSEA), Immune infiltration and microenvironments analysis were used to analyze the function of key genes. Machine learning selected four key OXPHOS-related genes associated with MMD: *CSK*, *NARS2*, *PTPN6* and *SMAD2* (*PTPN6* was upregulated and the other three were downregulated).

**Results:** Functional enrichment analysis showed that these genes were mainly enriched in the Notch signaling pathway, GAP junction, and RNA degradation, which are related to several biological processes, including angiogenesis, proliferation of vascular smooth muscle and endothelial cells, and cytoskeleton regulation. Immune analysis revealed immune infiltration and microenvironment in these MMD samples and their relationships with four key OXPHOS-related genes. APC co-inhibition (*p* = 0.032), HLA (*p* = 0.001), MHC I (*p* = 0.013), T cellco- inhibition (*p* = 0.032) and Type I IFN responses (*p* < 0.001) were significantly higher in the MMD groups than those in the control groups. The *CSK* positively correlated with APC co-inhibition and T cell-co-inhibition. The *NARS2* negatively correlated with Type I IFN response. The *SMAD2* negatively correlated with APC co-inhibition and Type I IFN response. The *PTPN6* positively correlated with HLA, MHC I and Type I IFN responses.

**Discussion:** This study provides a comprehensive understanding of the role of OXPHOS in MMD and will contribute to the development of new treatment methods and exploration of MMD pathogenesis.

## 1 Introduction

Moyamoya Disease (MMD) is a cerebrovascular disease characterized by chronic progressive occlusion of the distal end of the internal carotid artery or the main branches of the Willis circle. This occlusion causes the compensatory proliferation of small blood vessels at the brain base, presented as a ‘smoke-like’ vascular network on digital subtraction angiography (DSA) (Gonzalez et al., 2023). MMD may have catastrophic consequences such as hemorrhagic or ischemic stroke. In addition, it can cause serious symptoms such as motor dysfunction, sensory dysfunction, cognitive disorders, and epilepsy (Liu et al., 2023; Fang et al., 2024). However, its pathogenesis remains unclear. Currently, intracranial revascularization surgery is the primary treatment for MMD (Ihara et al., 2022). Although several targets related to MMD pathogenesis, such as *RNF213* and *DIAPH1*, have been discovered recently, corresponding drugs have not been developed based on these targets (He et al., 2023a; Fang et al., 2024). Few effective drugs for treating MMD are available because of the unknown molecular mechanisms underlying its occurrence (Ihara et al., 2022). Therefore, the mechanism of MMD should be urgently clarified to improve treatments.

Oxidative phosphorylation (OXPHOS), a crucial biochemical process in eukaryotic cells, is the final stage of aerobic respiration, followed by glycolysis and citric acid cycle (Wilson, 2017). It reduces oxygen to generate high-energy phosphate bonds in the form of adenosine triphosphate (ATP) and is the most efficient stage in the electron transport chain to generate ATP (Nolfi-Donegan et al., 2020). The ATP generated through OXPHOS is the primary energy source for cells and plays a crucial role in various physiological and pathological cellular processes (Lei et al., 2017). Recently, numerous studies have investigated the role of OXPHOS in vascular diseases. A previous study has reported that CHCHD4 orchestrates mitochondrial OXPHOS and antagonizes the aberrant growth and migration of pulmonary artery smooth muscle cells in pulmonary arterial hypertension (PAH) (Wang et al., 2023). The glutamine antagonist 6-diazo-5-oxo-L-norleucine (DON) inhibits vascular smooth muscle cell (VSMC) proliferation and migration by attenuating OXPHOS in atherosclerosis (Park et al., 2021). OXPHOS is essential for purinergic receptor-mediated angiogenic responses in vascular endothelial cells (ECs) (Lapel et al., 2017). Pathological mutations in the OXPHOS system subunits can increase vascular endothelial growth factor (VEGF) production and pathological angiogenesis (Bayona-Bafaluy et al., 2019). Thus, OXPHOS plays an important role in vascular disease pathogenesis.

Previous studies have demonstrated that OXPHOS is related to abnormal EC, VSMC, and angiogenesis, which might play important roles in vascular disease pathogenesis. As a cerebral vascular disease, the pathological characteristics of MMD also includes abnormal EC proliferation, abnormal VSMC migration, pathological angiogenesis, and vascular remodeling (Takagi et al., 2016; He et al., 2023b; He et al., 2023c). This generates evidence that OXPHOS disorder or regulation is closely related to MMD mechanism. However, studies on the relationship between MMD and OXPHOS remain scarce. Although a previous study has mentioned their relationship (Kanamori et al., 2021), further exploration is needed to study the role of OXPHOS in MMD, such as signaling pathways, related genes, and the immune response. Therefore, investigating the association between OXPHOS and MMD is urgently needed.

In this study, we collected MMD-related gene expression datasets and OXPHOS-related gene sets from Gene Expression Omnibus (GEO) and Genecards database. Machine learning algorithms of Least absolute shrinkage and selection operator (LASSO) and support vector machine-recursive feature elimination (SVM-RFE) were utilized to selected the key OXPHOS-genes related to MMD. We finally identified 4 key OXPHOS-related genes, *CSK*, *NARS2*, *PTPN6*, and *SMAD2*, and analyzed their function by KEGG and GO enrichment analysis, which showed that these genes were most relevant to MMD pathogenesis. GSEA analysis were also performed to provide the profile of immune infiltration and microenvironment about these genes. Ultimately, we identified the associations between these 4 key OXPHOS-related genes and the pathogenesis of MMD.

## 2 Materials and methods

### 2.1 Data acquisition

Gene expression data were retrieved from RNA sequencing data from the GEO database (https://www.ncbi.nlm.nih.gov/geo/info/datasets.html). The series matrix data files GSE189993, GSE157628, and GSE141024 enrolled 32, 20, and 8 patients, respectively, including 11, 9, and 4 in the control group, respectively, and 21, 9, and 4 in the MMD group, respectively. All the series matrix files were annotated using the GPL16699 file. OXPHOS-related gene sets were downloaded in the GeneCards database (https://www.genecards.org/). Those OXPHOS-related genes include protein-coding genes, functional element genes and RNA genes. The relevance score of GeneCards is a comprehensive score that measures the correlation between genes and specific keywords or topics based on multiple data sources and algorithms. Choosing a higher reliability score threshold can reduce the interference of noise and unrelated genes, enhance the reliability of analysis results, and ensure that the number of genes is not too large. Referring to several previous studies about screening genes with specific functions in GeneCards dataset (Li et al., 2022; Liu et al., 2022), we set the threshold of relevance score >15 to ensure that the selected genes have sufficient correlation and potential biological significance of oxidative phosphorylation.

### 2.2 Gene differential expression analysis

After merging the three datasets, the correction of the microarray data was performed using a surrogate variable analysis (SVA) package to reduce batch effects among them. Subsequently, we used principal component analysis (PCA) to draw a sample distribution map before and after correction, visually demonstrating the changes in batch effects before and after correction. The R package ‘limma’ was utilized to identify the differentially expressed genes (DEGs) between MMD and control samples by analyzing the gene expression data from the GEO cohort. Due to the small sample size in this study, *p*-value <0.05 was set as the criteria for filtering the DEGs to reduce the omissions of some potential biologically significant genes, rather using padj or FDR value. Meanwhile, the |log2 Fold Change (FC)| >1 was also set as the criteria for DEGs identification combined with *p* < 0.05 to ensure that the screened genes have significant differences in expression levels. Therefore, the final criteria of filtering DEGs was set as *p* < 0.05 and |log2 FC| >1, which was also be utilized in previous studies (Liu et al., 2020; Wang et al., 2022).

### 2.3 GO and KEGG functional enrichment analysis

GO and KEGG enrichment analyses were performed to evaluating the biological function, biological process, molecular function, and cellular component of the intersection genes between DEGs and OXPHOS-related gene set. When performing the enrichment analysis, we utilized the R package ‘clusterProfiler’ to perform the GO and KEGG enrichment analysis. The default threshold for ‘clusterProfiler’ package to do enrichment analysis is a *p*-value <0.05 and a Q value <0.2. In this study, to make the enrichment results more accurate, we further limited the Q value as less than 0.05, which is a relative strict standard. Finally, we set the cutoffs of GO and KEGG enrichment analysis as a *p*-value <0.05 and a Q value <0.05, referring to several previous studies (Liu et al., 2023; Li et al., 2024).

### 2.4 Key genes and possible disease diagnostic indicator selection

LASSO logistic regression and SVM-RFE algorithm were used to select the potential disease diagnostic markers. LASSO logistic regression was conducted using ‘glmnet’ package. When performing LASSO logistic regression, we used the ‘glmnet’ package to fit gene expression levels based on the glmnet function, with the family set as gaussian distribution, and automatically selects the optimal lambda value. By using the ‘cv.glmnet’ function for 10 fold cross validation, the optimal regularization parameter lambda was selected, and the performance curves of the model were plotted for each lambda value during the cross validation process. Finally, we selected the optimal lambda value by observing the lowest point of the curve. SVM-RFE machine learning model was constructed using ‘e1071’ package to further identify the significance of indicators for disease diagnosis. When performing SVM-RFE, we first used the SVM-RFE method to perform feature selection on DEGs to obtain the ranking of each gene. SVM model was established using the “e1071”R package, and the SVM model is constructed based on the top 10 genes to evaluate the lowest error rate of the model. The gene sets with the error rate reaching the lowest point and the genes obtained by the LASSO were intersected to obtain the key genes.

### 2.5 Gene set enrichment pathway analysis

Gene set enrichment analysis (GSEA) was used to investigate the DEGs between the high- and low-expression groups based on the expression profiles of patients with MvMD. GSEA was performed using a predefined gene set ‘c2. cp. Kegg’ to rank them according to their differential expression levels in the two types of samples. The preset gene sets were examined to determine whether they were enriched at the top or bottom of the ranking table. This study used GSEA software (http://www.broadinstitute.org/gsea) to compare the differences in KEGG signaling pathways between high expression and low expression groups, and explored the molecular mechanisms of key genes in the two groups of patients. The times of substitutions was set as 1,000 and the type of substitution was set as ‘phenotype.’ Pathways with a *p*-value <0.05 were considered significantly enriched.

### 2.6 Immune cell infiltration analysis

Single-sample GSEA (ssGSEA) was performed to evaluate the immune cells types in the microenvironment using ‘GSVA’ R package. We performed ssGSEA to quantify the immune cells in the expression profile and calculated the relative proportions of 19 types of infiltrating immune cells, including activated Dendritic Cells (aDCs), Antigen-Presenting Cells co-inhibition (APC_co_inhibition), Antigen-Presenting Cells co-stimulation (APC_co_stimulation), B lymphocytes (B_cells), C-C Chemokine Receptor (CCR), Checkpoint, Human Leukocyte Antigen (HLA), Inflammation-promoting, Macrophages, Major Histocompatibility Complex class I (MHC_class_I), Neutrophils, Parainflammation, plasmacytoid Dendritic Cells (pDCs), T cell co-inhibition, T follicular helper cells (Tfh), Tumor-Infiltrating Lymphocytes (TIL), Regulatory T cells (Treg), Type I Interferon Response (Type_I_IFN_Reponse) and Type II Interferon Response (Type_II_IFN_Reponse). Spearman’s correlation analysis was used to analyze the gene expression and immune cell content. *p* < 0.05 was considered statistically significant.

### 2.7 Statistical analysis

All statistical analyses were performed using the R software (version 4.2.2) and the figures were plotted using the ‘ggplot’ R package (version 3.3.6). Statistical significance was set at *p* < 0.05.

## 3 Results

### 3.1 DEG identification and functional enrichment analysis in the MMD cohort

**FIGURE 1:**
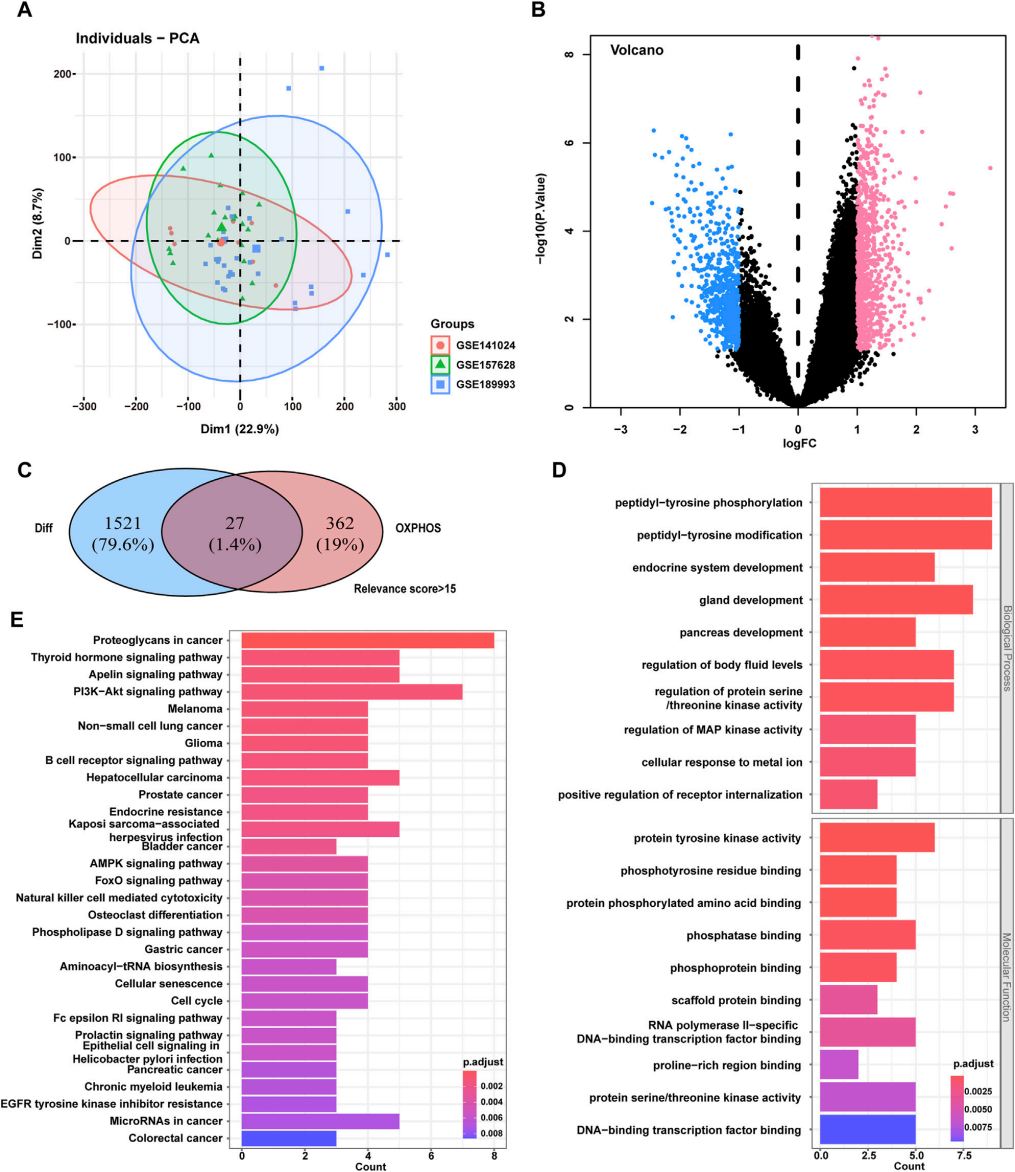
Screening of the phosphorylation-associated genes for MMD and functional enrichment. **(A)** PCA plot for gene expression data of the participants. **(B)** Volcano plot of DEGs differentially expressed between MMD patients and control participants in the GEO dataset. **(C)** Venn plot of the overlapping DEGs and phosphorylation-associated genes. Finally, 27 intersecting genes were selected. **(D)** GO enrichment analysis for the 27 intersecting genes. E, KEGG enrichment analysis for the 27 intersecting genes. MMD, Moyamoya disease; PCA, Principal component analysis; GEO, Gene Expression Omnibus; DEGs, Differentially expressed genes; GO, Gene Ontology; KEGG, Kyoto Encyclopedia of Genes and Genomes.

The MMD-related datasets GSE189993, GSE157628, and GSE141024 were downloaded from the GEO database and used in this study, including 60 samples from patients with MMD (n = 36) and control participants (n = 24). The detailed clinical information for the participants was listed in Supplementary Table S1 in Supplementary Material. Correction was performed using the SVA algorithm to reduce the batch effect between microarrays. The PCA plot shows that this process reduced the batch effect (Figure 1A).

A total of 1,548 genes were differentially expressed between patients with MMD and control participants (850 were upregulated and 698 were downregulated in the MMD group), according to *p* < 0.05 and |logFC|>1. The volcano plot displays an overview of the DEGs between patients with MMD and control participants (Figure 1B). The detailed information of these 1,548 DEGs were listed in Supplementary Table S2 in Supplementary Material. A total of 389 OXPHOS-related genes with >15 relevance score was screened from OXPHOS-associated gene sets in the GeneCards database (https://www.genecards.org/). The detailed information of these 389 OXPHOS-related genes were listed in Supplementary Table S3 in Supplementary Material. Finally, 27 intersecting genes were selected from the screened OXPHOS-associated genes and genes that were differentially expressed between the patients with MMD and control participants (Figure 1C). The 27 intersecting genes were also listed in Supplementary Table S3 in Supplementary Material.

GO biological process enrichment analysis revealed that these 27 genes were mainly enriched in peptidyl tyrosine phosphorylation, peptidyl tyrosine modification, and gland development. GO molecular function enrichment analysis revealed that these genes were mainly enriched in protein tyrosine kinase activity, phosphotyrosine residue binding and phosphatase binding (Figure 1D). The GO cellular component enrichment was not significant. KEGG enrichment analysis showed that these genes were mainly enriched in the apelin signaling pathway, PI3K–Akt signaling pathway, and endocrine resistance (Figure 1E).

### 3.2 Identification of key phosphorylation-associated DEGs by machine learning

**FIGURE 2:**
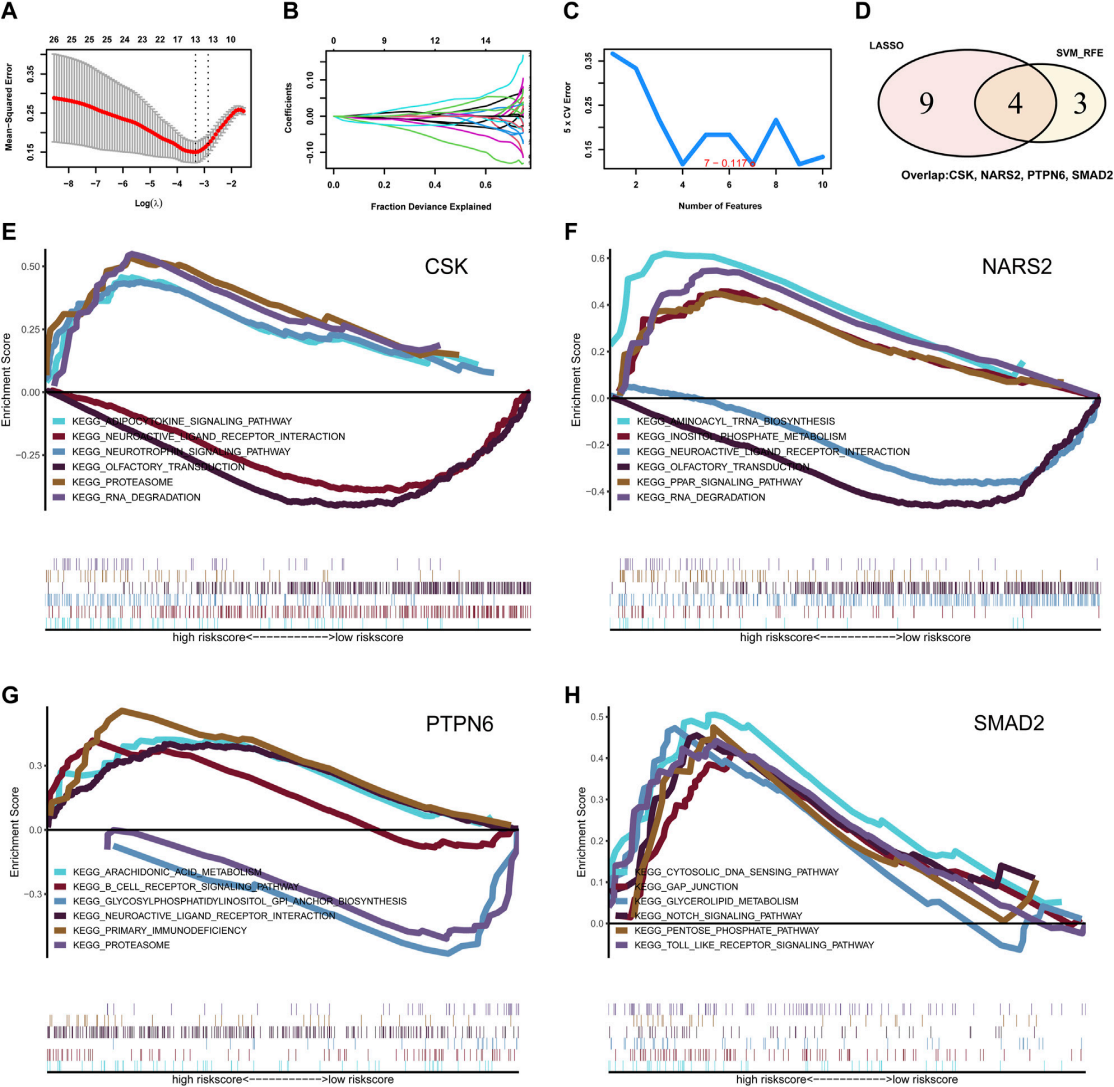
Identification for the key phosphorylation-associated genes for MMD by machine learning algorithm and functional enrichment. **(A, B)** Identification for characteristic genes for MMD by LASSO regression algorithm. Finally, 13 intersecting genes were selected. **(C)** Identification for characteristic genes for MMD by SVM algorithm. Finally, 7 intersecting genes were selected. **(D)** Venn plot of the overlapping genes identified by LASSO regression and SVM algorithm. Finally, 4 intersecting genes were selected as key phosphorylation-associated genes for MMD. **(E)** GSEA analysis for gene CSK. **(F)** GSEA analysis for gene NARS2. **(G)** GSEA analysis for gene PTPN6. **(H)** GSEA analysis for gene SMAD2. MMD, Moyamoya disease; LASSO, Least Absolute Shrinkage and Selection Operator; SVM, Support Vector Machine; GSEA, Gene set enrichment analysis.

To further identify the key genes affecting MMD, LASSO logistic regression and SVM algorithms were performed based on the previously obtained intersecting genes. The results identified 13 genes as MMD characteristic genes using LASSO regression (Figures 2A,B). We also used the SVM algorithm to identify characteristic genes for MMD and screened the top seven genes with the lowest error rates (Figure 2C). The genes screened by LASSO regression and the SVM algorithm were then intersected, and four genes were selected, including three downregulated genes, *CSK*, *NARS2*, and *PTPN6*, and one upregulated gene, *SMAD2* (Figure 2D). These four genes were considered key genes in MMD and further investigated.

### 3.3 Functional enrichment analysis for the key genes in MMD

Next, we investigated the signaling pathways enriched in these four genes and explored the potential molecular mechanisms affecting MMD progression. GSEA results showed that *CSK* was enriched in the adipocytokine signaling pathway, neurotrophin signaling pathway, proteasome, neuroactive ligand–receptor interaction, olfactory transduction, and RNA degradation (Figure 2E). *NARS2* was enriched in aminoacyl-tRNA biosynthesis, inositol phosphate metabolism, neuroactive ligand–receptor interaction, olfactory transduction, PPAR signaling pathway, and RNA degradation (Figure 2F). *PTPN6* was enriched in arachidonic acid metabolism, the B cell receptor signaling pathway, glycosylphosphatidylinositol (GPI) anchor biosynthesis, neuroactive ligand–receptor interaction, primary immunodeficiency, and proteasomes (Figure 2G). *SMAD2* was enriched in the cytosolic NDA sensing pathway, GAP junction, glycerolipid metabolism, Notch signaling pathway, pentose phosphate pathway, and toll-like receptor signaling pathway (Figure 2H).

### 3.4 Analysis of immune infiltration and microenvironment

**FIGURE 3:**
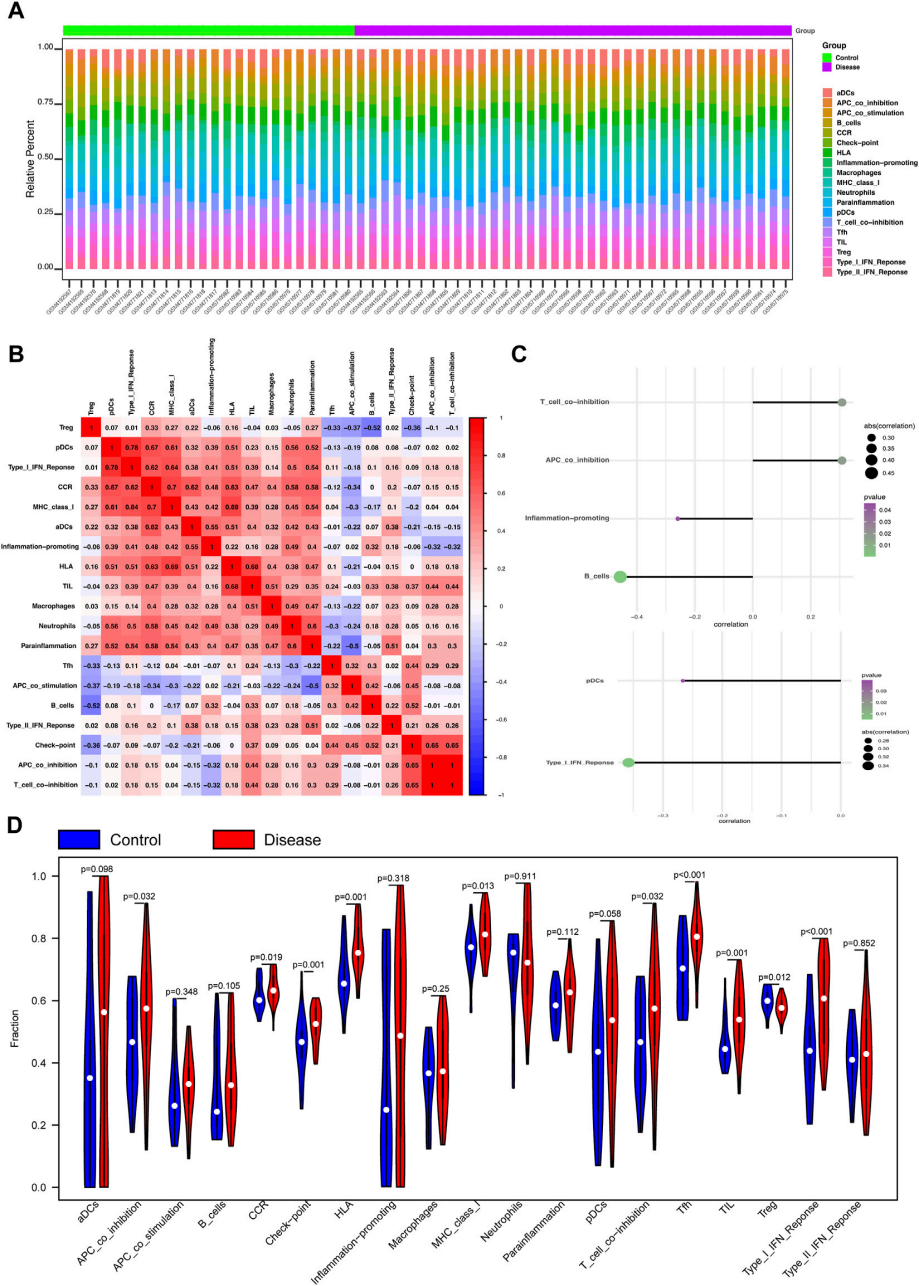
Analysis of immune infiltration and microenvironment of the four key phosphorylation-associated genes for MMD. **(A)** Heatmap for the proportion of 19 kinds of immune cells in each sample. **(B)** Thermodynamic plot of the relationship between each kind of immune cells. **(C)** The relationship between the gene CSK (upper of the figure) and NARS2 (lower of the figure), and immune cells. **(D)** Violin plot for the difference of immune infiltration between MMD and control group. MMD = Moyamoya disease.

Next, the correlation between the four key genes and immune cell infiltration was analyzed. The heatmap shows the proportion of 19 immune cell types in each sample (Figure 3A). A thermodynamic chart displays the relationship with each immune cell type (Figure 3B). The results showed that APC co-inhibition (*p* = 0.032), CCR (*p* = 0.019), checkpoint (*p* = 0.001), HLA (*p* = 0.001), MHC I (*p* = 0.013), T cell-co-inhibition (*p* = 0.032), TFH cells (*p* < 0.001), TIL cells (*p* = 0.001), Treg cells (*p* = 0.012), and Type I IFN responses (*p* < 0.001) were significantly higher in the MMD groups than those in the control groups (Figure 3D).

Next, we analyzed the relationships between the four key genes and immune cells. The results showed that *CSK* significantly positively correlated with T cell co-inhibition and APC co-inhibition, and significantly negatively correlated with B cells and inflammation promotion (Figure 3C). *NARS2* significantly negatively correlated with pDCs and the Type I IFN response (Figure 3C). *SMAD2* positively correlated with macrophages, but significantly negatively correlated with TFH cells, APC co-inhibition, pDCs, and the Type I IFN response (Supplementary Figure S1F). *PTPN6* positively correlated with TIL, CCR, macrophages, HLA, neutrophil MHC I, and inflammation (Supplementary Figure S1F). Bubble plots showed the relationship between the four key genes and the immune microenvironment, including chemokine, immunoinhibitor, receptor, immunostimulatory, and MHC (Supplementary Figures S1A–E).

### 3.5 In depth analysis of the correlation between the key genes and MMD

**FIGURE 4:**
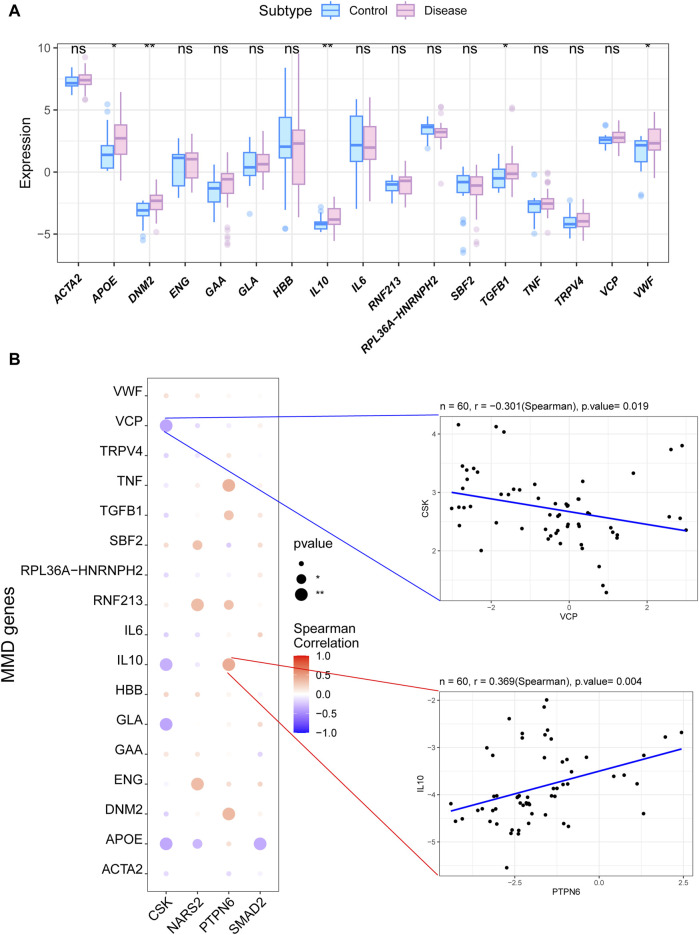
Analysis of the correlation between the key genes and MMD. **(A)** Box plot for the expression levels of DEGs for MMD related to the key phosphorylation-associated genes. **(B)** Bubble plot for the relationship between DEGs for MMD and the key phosphorylation-associated genes. MMD = Moyamoya disease; DEGs = Differentially expressed genes.

To further explore the potential molecular mechanisms of the four key genes in MMD, we analyzed other related genes. We first analyzed differences in the expression of MMD-related genes obtained from the GeneCards database between the MMD and control groups. The results showed that *APOE*, *DNM2*, *IL10*, *TGFβ1*, and *VWF* expression levels in MMD group were significantly higher than that in control group (Figure 4A). Next, we analyzed the correlation between MMD-related genes and these four key genes. Significant correlations were observed among these variables. Among these, *PTPN6* was significantly and positively correlated with *DNM2*, whereas *CSK* was significantly and negatively correlated with *APOE* (Figure 4B).

## 4 Discussion

A considerable proportion of patients with MMD may suffer from serious cerebral hemorrhage or ischemic stroke; thus, timely intervention is required. Currently, intracranial revascularization surgery is the main treatment for treating MMD (Ihara et al., 2022). Due to possible postoperative complications, such as stroke or infection, surgery cannot achieve a satisfactory prognosis in some patients with MMD (Kuroda and Houkin, 2008; Liu et al., 2023). Therefore, novel treatment methods should be urgently developed. However, owing to the unclear mechanism of MMD, there is a lack of effective drugs to prevent its progression, as well as effective animal models (Rallo et al., 2021; Ihara et al., 2022). Although some biological targets have recently been identified by genomics, proteomics, or other methods, animal modeling and drug research based on these targets have achieved unsatisfactory results (Rallo et al., 2021; Ihara et al., 2022). This suggests that other mechanisms may be involved in MMD development. Several studies have reported the role of OXPHOS in vascular diseases (Lapel et al., 2017; Bayona-Bafaluy et al., 2019; Park et al., 2021; Wang et al., 2023), indicating that OXPHOS may also be involved in MMD pathogenesis. However, few studies have examined the relationship between OXPHOS and MMD. Therefore, in this study, we systematically investigated the role of OXPHOS-related genes, signaling pathways, and immune responses in the pathogenesis of MMD using bioinformatics. Four key genes were selected: *CSK*, *NARS2*, *PTPN6*, and *SMAD2*.

*SMAD2* is a signal transducer and transcriptional modulator that mediates multiple signaling pathways (Nomura and Li, 1998). This protein mediates transforming growth factor beta (TGFβ) signal, and thus regulates multiple cellular processes, such as cell proliferation, apoptosis, and differentiation (Kamato and Little, 2020). In our study, we found a significantly downregulation of *SMAD2* expression. As for previous studies of *SMAD2*, Petersen et al. found that *SMAD2* knockdown developed an aggressive phenotype in breast cancer cells and enhanced angiogenesis (Petersen et al., 2010). Cao et al. found that activin receptor-like kinase 7 promotes VSMC apoptosis by activating the Smad2/3 signaling in diabetic atherosclerosis (Cao et al., 2022). These studies provided evidence that *SMAD2* downregulation in patients with MMD might promote angiogenesis, proliferation and phenotype switching of VSMCs, which are the important characteristics of MMD mentioned before (Ma et al., 2022; He et al., 2023c; He et al., 2023d). As for the results in our study, pathway enrichment analysis also showed that *SMAD2* was enriched in the Notch, GAP junction, and toll-like receptor signaling pathways, which are also associated with angiogenesis, vascular development, endothelial cell and VSMC growth in previous studies (Beyer and Berthoud, 2018; Shafeghat et al., 2022; Cuervo et al., 2023). In summary, these results generated the evidence that *SMAD2* may play an important role in MMD pathogenesis and vascular changes.

*CSK* is a negative regulator of Src family kinases (SFKs) recruited to the plasma membrane by binding to transmembrane or adapter proteins to suppress signaling via various surface receptors by phosphorylating and maintaining several inactive positive effectors, such as FYN or LCK (D and CJB, 1999; Sun and Ayrapetov, 2023). It plays important roles in cell growth, migration, differentiation, and immune response (Zhu et al., 2023). There were several studies about the *CSK* on endothelial cells. Alejandro et al. found that *CSK* activation renders the endothelial barrier more susceptible to TNF-α through p38 activation, which leads to endothelial tight junction loss (Adam et al., 2016). Ulf et al. reported that *CSK* is involved in endothelial cell proliferation inhibition (Baumeister et al., 2005). Previous studies showed that the *CSK* has an inhibitory effect on the proliferation of endothelial cells. In this study, we found a downregulated of *CSK* expression in the MMD group. Accordingly, we inferred that may promote the angiogenesis through stimulating the endothelial cell proliferation, which may cause a intimal thickening of MMD vessels reported in previous study (Takagi et al., 2016; He et al., 2023b).

*NARS2* encodes asparaginyl-tRNA synthetase 2, a putative member of the class II family of aminoacyl-tRNA synthetases. This enzyme catalyzes asparagine attachment to mitochondrial tRNA and plays a critical role in mitochondrial protein biosynthesis (Duchene et al., 2009). *NARS2* mutations are associated with a combined oxidative phosphorylation deficiency 24 and autosomal recessive deafness-94 (Sissler et al., 2017). In this study, *NARS2* was downregulated but there is a lack of research about the relationship between *NARS2* and MMD. Due to its association with cellular energy metabolism, we inferred that *NARS2* downregulation in MMD may lead to a deficiency in mitochondrial protein synthesis and affect the energy supply within the cell, leading to VSMC and EC dysfunction in MMD vessels.

*PTPN6* is a member of the protein tyrosine phosphatase (PTP) family, a group of enzymes that remove phosphate groups from tyrosine residues by hydrolyzing phosphoric acid monoesters. PTPs are signaling molecules that regulate several cellular processes, including cell growth, differentiation, mitotic cycle, and oncogenic transformation (Amin et al., 2021). In this study, *PTPN6* was the only upregulated among the four key genes. In previous studies, Clement et al. found that *PTPN6* overexpression fully restored PDGF-induced proliferation, migration, and signaling pathways in SMC exposed to high glucose and hypoxia (Clément et al., 2021). As for the role of VSMC in MMD, a previous study reported VSMC over-proliferation in the intima and intimal thickening of pathological blood vessels in MMD (Takekawa et al., 2004). This generates evidence that *PTPN6* overexpression in MMD may promote VSMC proliferation and subsequently cause intimal thickening. In addition, *PTPN6* expression was significantly positively correlated with the expression of dynamin 2 (*DNM2*) in this study, a GTP-binding protein subfamily and an important component of the cellular cytoskeleton (Ferguson and De Camilli, 2012). Interestingly, our previous study also found that cytoskeletal remodeling in ECs plays an important role in MMD pathogenesis (He et al., 2023b). This suggests that *PTPN6* and *DNM2* are involved in MMD by participating in regulating the cellular cytoskeleton.

Our study has limitations. First, the sample size collected in this study was relatively small, which may have reduced the representativeness of the study and weaken the level of evidence when drawing conclusions about causality between identified genes and MMD pathogenesis. Further study needs to be conducted in a larger and ethnically diverse patient population to strengthen the generalizability of the findings. Second, the key genes and their functions in this study are lack of validation through *in vitro* experiments. Functional experiments are needed to validate the impact of the identified genes on relevant cellular processes in MMD, for example, the cell proliferation assays or migration assays on the gene overexpression or knockdown cell lines. Finally, in order to avoid the omissions of some potential biologically significant genes in the small sample size, this study used the criteria of *p* < 0.05 and | log2FC | > 1 to identify DEGs, without using adjusted *p*-values or FDR values. This method may lead to a higher false positive rate, which is also a limitation of this study.

## 5 Conclusion

In summary, this study identified four key OXPHOS-related genes associated with MMD: *CSK*, *NARS2*, *PTPN6*, and *SMAD2*. We systematically investigated the relationship between four key OXPHOS-related genes and the pathogenesis of MMD We also analyzed the profile of immune infiltration and the microenvironment of these genes. Bioinformatic analysis showed that the four key genes are related with angiogenesis, endothelial cell and VSMC proliferation, and VSMC phenotype switching, which may be involved in the mechanisms of MMD. Further studies will be needed to investigate the role of these identified genes in the pathogenesis of MMD in a larger and ethnically diverse patient population, or by *in vivo* and *in vitro* experiments. In conclusion, this study provides a comprehensive understanding of the important role of OXPHOS in MMD pathogenesis and will be conducive to the development of new treatment methods for MMD.

## Data Availability

The data presented in the study are deposited in the GEO repository (https://www.ncbi.nlm.nih.gov/geo/info/datasets.html), accession number GSE189993, GSE157628, GSE141024.

## References

[B1] Adam A. P. Lowery A. M. Martino N. Alsaffar H. Vincent P. A. (2016). Src family kinases modulate the loss of endothelial barrier function in response to TNF-α: crosstalk with p38 signaling. PLoS One 11 (9), e0161975. 10.1371/journal.pone.0161975 27603666 PMC5014308

[B2] Amin A. D. S. Chizuru A. Hajjaj H. M. A.-A. Suganuma Y. Imamura A. Ando H. (2021). The protein tyrosine phosphatase SHP-1 (PTPN6) but not CD45 (PTPRC) is essential for the ligand-mediated regulation of CD22 in BCR-ligated B cells. J. Immunol. 206, 2544–2551. 10.4049/jimmunol.2100109 33990399

[B3] Baumeister U. Funke R. Ebnet K. Vorschmitt H. Koch S. Vestweber D. (2005). Association of Csk to VE-cadherin and inhibition of cell proliferation. EMBO J. 24 (9), 1686–1695. 10.1038/sj.emboj.7600647 15861137 PMC1142580

[B4] Bayona-Bafaluy M. Esteban O. Ascaso J. Montoya J. Ruiz-Pesini E. (2019). Oxidative phosphorylation inducers fight pathological angiogenesis. Drug Discov. Today 24 (9), 1731–1734. 10.1016/j.drudis.2019.03.014 30880173

[B5] Beyer E. C. Berthoud V. M. (2018). Gap junction gene and protein families: connexins, innexins, and pannexins. Biochimica Biophysica Acta (BBA) - Biomembr. 1860 (1), 5–8. 10.1016/j.bbamem.2017.05.016 PMC5704981 28559187

[B6] Cao S. Yuan Q. Dong Q. Liu X. Liu W. Zhai X. (2022). Activin receptor-like kinase 7 promotes apoptosis of vascular smooth muscle cells via activating Smad2/3 signaling in diabetic atherosclerosis. Front. Pharmacol. 13, 926433. 10.3389/fphar.2022.926433 36059980 PMC9428160

[B7] Clément M. Tristan B. Jérémy L. Boisvert E. Robillard S. Breton V. (2021). Diabetes impaired ischemia-induced PDGF (Platelet-Derived growth factor) signaling actions and vessel formation through the activation of scr homology 2-containing phosphatase-1. Arterioscler. Thromb. Vasc. Biol. 41, 2469–2482. 10.1161/ATVBAHA.121.316638 34320834

[B8] Cuervo H. Muhleder S. Garcia-Gonzalez I. Benedito R. (2023). Notch-mediated cellular interactions between vascular cells. Curr. Opin. Cell Biol. 85, 102254. 10.1016/j.ceb.2023.102254 37832167

[B9] D S. Cjb P. A. (1999) Domain interactions in protein tyrosine kinase Csk, 38. 10.1021/bi990827+ 10460171

[B10] Duchene A. M. Pujol C. Marechal-Drouard L. (2009). Import of tRNAs and aminoacyl-tRNA synthetases into mitochondria. Curr. Genet. 55 (1), 1–18. 10.1007/s00294-008-0223-9 19083240

[B11] Fang J. Yang X. Ni J. (2024). RNF213 in moyamoya disease: genotype-phenotype association and the underlying mechanism. Chin. Med. J. Engl. 10.1097/CM9.0000000000002985 PMC11557053 38243713

[B12] Ferguson S. M. De Camilli P. (2012). Dynamin, a membrane-remodelling GTPase. Nat. Rev. Mol. Cell Biol. 13 (2), 75–88. 10.1038/nrm3266 22233676 PMC3519936

[B13] Gonzalez N. R. Amin-Hanjani S. Bang O. Y. Coffey C. Du R. Fierstra J. (2023). Adult moyamoya disease and syndrome: current perspectives and future directions: a scientific statement from the American heart association/American stroke association. Stroke 54 (10), e465–e479. 10.1161/STR.0000000000000443 37609846

[B14] He S. Hao X. Liu Z. Wang Y. Zhang J. Wang X. (2023a). Association between DIAPH1 variant and posterior circulation involvement with Moyamoya disease. Sci. Rep. 13 (1), 10732. 10.1038/s41598-023-37665-1 37400591 PMC10318013

[B15] He S. Liang J. Xue G. Wang Y. Zhao Y. Liu Z. (2023d). RNA profiling of sEV (small extracellular vesicles)/exosomes reveals biomarkers and vascular endothelial dysplasia with moyamoya disease. J. Cereb. Blood Flow. Metab. 43 (7), 1194–1205. 10.1177/0271678X231162184 36883376 PMC10291455

[B16] He S. Wang Y. Liu Z. Zhang J. Hao X. Wang X. (2023c). Metabolomic signatures associated with pathological angiogenesis in moyamoya disease. Clin. Transl. Med. 13 (12), e1492. 10.1002/ctm2.1492 38037492 PMC10689969

[B17] He S. Zhang J. Liu Z. Wang Y. Hao X. Wang X. (2023b). Upregulated cytoskeletal proteins promote pathological angiogenesis in moyamoya disease. Stroke 54 (12), 3153–3164. 10.1161/STROKEAHA.123.044476 37886851

[B18] Ihara M. Yamamoto Y. Hattori Y. Liu W. Kobayashi H. Ishiyama H. (2022). Moyamoya disease: diagnosis and interventions. Lancet Neurol. 21 (8), 747–758. 10.1016/S1474-4422(22)00165-X 35605621

[B19] Kamato D. Little P. J. (2020). Smad2 linker region phosphorylation is an autonomous cell signalling pathway: implications for multiple disease pathologies. Biomed. Pharmacother. 124, 109854. 10.1016/j.biopha.2020.109854 31981946

[B20] Kanamori F. Yokoyama K. Ota A. Yoshikawa K. Karnan S. Maruwaka M. (2021). Transcriptome-wide analysis of intracranial artery in patients with moyamoya disease showing upregulation of immune response, and downregulation of oxidative phosphorylation and DNA repair. Neurosurg. Focus 51 (3), E3. 10.3171/2021.6.FOCUS20870 34469870

[B21] Kuroda S. Houkin KJTLN (2008). Moyamoya disease: current concepts and future perspectives. Lancet. Neurol. 7 (11), 1056–1066. 10.1016/S1474-4422(08)70240-0 18940695

[B22] Lapel M. Weston P. Strassheim D. Karoor V. Burns N. Lyubchenko T. (2017). Glycolysis and oxidative phosphorylation are essential for purinergic receptor-mediated angiogenic responses in vasa vasorum endothelial cells. Am. J. Physiol. Cell Physiol. 312 (1), C56–C70. 10.1152/ajpcell.00250.2016 27856430 PMC5283894

[B23] Lei D. Yi-Fa C. Peter J. C. Xiao-Ping CJCI. Extracellular ATP signaling and clinical relevance. 2017; 188. 10.1016/j.clim.2017.12.006 29274390

[B24] Li S. Sun Y. Sun Y. (2022). A comparative study of systems pharmacology and gene chip technology for predicting targets of a traditional Chinese medicine formula in primary liver cancer treatment. Front. Pharmacol. 13, 768862. 10.3389/fphar.2022.768862 35308212 PMC8926147

[B25] Li Z. Wei R. Yao S. Meng F. Kong L. (2024). HIF-1A as a prognostic biomarker related to invasion, migration and immunosuppression of cervical cancer. Heliyon 10 (2), e24664. 10.1016/j.heliyon.2024.e24664 38298716 PMC10828096

[B26] Liu L. Zhu Y. Fu P. Yang J. (2022). A network pharmacology based research on the mechanism of donepezil in treating alzheimer's disease. Front. Aging Neurosci. 14, 822480. 10.3389/fnagi.2022.822480 35462691 PMC9031729

[B27] Liu M. Saredy J. Zhang R. Shao Y. Sun Y. Yang W. Y. (2020). Approaching inflammation paradoxes—proinflammatory cytokine blockages induce inflammatory regulators. Front. Immunol. 11, 554301. 10.3389/fimmu.2020.554301 33193322 PMC7604447

[B28] Liu S. Xu H. Feng Y. Kahlert U. D. Du R. Torres-de la Roche L. A. (2023b). Oxidative stress genes define two subtypes of triple-negative breast cancer with prognostic and therapeutic implications. Front. Genet. 14, 1230911. 10.3389/fgene.2023.1230911 37519893 PMC10372428

[B29] Liu W. Huang K. Zhang J. Zhou D. Chen J. (2023a). Clinical features and risk factors of postoperative stroke in adult moyamoya disease. Brain Sci. 13 (12), 1696. 10.3390/brainsci13121696 38137144 PMC10741386

[B30] Ma X. Huang Y. He X. Zhang X. Liu Y. Yang Y. (2022). Endothelial cell–derived let-7c-induced TLR7 activation on smooth muscle cell mediate vascular wall remodeling in moyamoya disease. Transl. Stroke Res. 14 (4), 608–623. 10.1007/s12975-022-01088-3 36181627

[B31] Nolfi-Donegan D. Braganza A. Shiva S. (2020). Mitochondrial electron transport chain: oxidative phosphorylation, oxidant production, and methods of measurement. Redox Biol. 37, 101674. 10.1016/j.redox.2020.101674 32811789 PMC7767752

[B32] Nomura M. Li E. J. N. Smad2 role in mesoderm formation, left-right patterning and craniofacial development., 1998; 393(6687): 786–790. 10.1038/31693 9655392

[B33] Park H. Y. Kim M. J. Lee S. Jin J. Kim J. G. (2021). Inhibitory effect of a glutamine antagonist on proliferation and migration of VSMCs via simultaneous attenuation of glycolysis and oxidative phosphorylation. Int. J. Mol. Sci. 22 (11), 5602. 10.3390/ijms22115602 34070527 PMC8198131

[B34] Petersen M. Pardali E. van der Horst G. Cheung H. van den Hoogen C. van der Pluijm G. (2010). Smad2 and Smad3 have opposing roles in breast cancer bone metastasis by differentially affecting tumor angiogenesis. Oncogene 29 (9), 1351–1361. 10.1038/onc.2009.426 20010874

[B35] Rallo M. Akel O. Gurram A. Sun H. (2021). Experimental animal models for moyamoya disease and treatment: a pathogenesis-oriented scoping review. Neurosurg. Focus 51 (3), E5. 10.3171/2021.6.FOCUS21284 34469865

[B36] Shafeghat M. Kazemian S. Aminorroaya A. Aryan Z. Rezaei N. (2022). Toll-like receptor 7 regulates cardiovascular diseases. Int. Immunopharmacol. 113, 109390. 10.1016/j.intimp.2022.109390 36330918

[B37] Sissler M. Gonzalez-Serrano L. E. Westhof E. (2017). Recent advances in mitochondrial aminoacyl-tRNA synthetases and disease. Trends Mol. Med. 23 (8), 693–708. 10.1016/j.molmed.2017.06.002 28716624

[B38] Sun G. Ayrapetov M. K. (2023). Dissection of the catalytic and regulatory structure-function relationships of Csk protein tyrosine kinase. Front. Cell Dev. Biol. 11, 1148352. 10.3389/fcell.2023.1148352 36936693 PMC10016382

[B39] Takagi Y. Hermanto Y. Takahashi J. C. Funaki T. Kikuchi T. Mineharu Y. (2016). Histopathological characteristics of distal middle cerebral artery in adult and pediatric patients with moyamoya disease. Neurol. Med. Chir. (Tokyo) 56 (6), 345–349. 10.2176/nmc.oa.2016-0031 27087193 PMC4908078

[B40] Takekawa Y. Umezawa T. Ueno Y. Sawada T. Kobayashi M. (2004). Pathological and immunohistochemical findings of an autopsy case of adult moyamoya disease. Neuropathology 24 (3), 236–242. 10.1111/j.1440-1789.2004.00550.x 15484702

[B41] Wang W. Liang Q. Zhao J. Pan H. Gao Z. Fang L. (2022). Low expression of the metabolism-related gene SLC25A21 predicts unfavourable prognosis in patients with acute myeloid leukaemia. Front. Genet. 13. 10.3389/fgene.2022.970316 PMC9562002 36246603

[B42] Wang Y. Zeng Z. Zeng Z. Chu G. Shan X. (2023). Elevated CHCHD4 orchestrates mitochondrial oxidative phosphorylation to disturb hypoxic pulmonary hypertension. J. Transl. Med. 21 (1), 464. 10.1186/s12967-023-04268-3 37438854 PMC10339524

[B43] Wilson D. F. (2017). Oxidative phosphorylation: regulation and role in cellular and tissue metabolism. J. Physiol. 595 (23), 7023–7038. 10.1113/JP273839 29023737 PMC5709332

[B44] Zhu S. Wang H. Ranjan K. Zhang D. (2023). Regulation, targets and functions of CSK. Front. Cell Dev. Biol. 11, 1206539. 10.3389/fcell.2023.1206539 37397251 PMC10312003

