# Supplementary material for: Identification of oxidative phosphorylation-related genes in moyamoya disease by combining bulk RNA-sequencing analysis and machine learning
Source: Front Genet. 2024 Jun 10;15:1417329. doi: 10.3389/fgene.2024.1417329 (PMC11197386; doi:10.3389/fgene.2024.1417329)
Supplement: Supplementary file 3 [file Table1.DOCX]

**Supplementary Materials**

**Table S1:** Detailed clinical information for the participants.

| ID | Disease | Age  (year) | Sex | Taken medicines | Antibiotics for the operation | Clinical presentation | Duration until surgery from the last clinical presentation | Subtype  of MMD | Aneurysm  location/size | Epileptic  origin | GEO database ID |
| --- | --- | --- | --- | --- | --- | --- | --- | --- | --- | --- | --- |
| 1 | MMD | 64 | F | Atorvastatin 10 mg/day | Cefazolin 1g | ICH | 2 months | Bilateral | - | - | GSE157628 |
| 2 | MMD | 47 | F | Aspirin 100 mg/day,  atorvastatin 10 mg/day | Ceftriaxone 2g | TIA | 5 months | Bilateral | - | - | GSE157628 |
| 3 | MMD | 48 | F | Amlodipine 5 mg/day | Ceftriaxone 2g | ICH | 6 months | Bilateral | - | - | GSE157628 |
| 4 | MMD | 49 | F | Aspirin 100 mg/day | Cefazolin 1g | TIA | 6 months | Bilateral | - | - | GSE157628 |
| 5 | MMD | 59 | F | Cilostazol 200 mg/day | Cefazolin 1g | IF | 11 months | Bilateral | - | - | GSE157628 |
| 6 | MMD | 50 | F | Cilostazol 200 mg/day | Cefazolin 1g | IF | 8 months | Bilateral | - | - | GSE157628 |
| 7 | MMD | 45 | F | Aspirin 100 mg/day,  clopidogrel 75 mg/day | Clindamycin  600 mg | TIA | 1 month | Bilateral | - | - | GSE157628 |
| 8 | MMD | 48 | F | Aspirin 100 mg/day | Cefazolin 1g | TIA | 4 months | Bilateral | - | - | GSE157628 |
| 9 | MMD | 51 | M | Aspirin 100 mg/day,  olmesartan 20 mg/day,  azelnidipine 16 mg/day, pitavastatin 2 mg/day | Cefazolin 1g | TIA | 2 months | Bilateral | - | - | GSE157628 |
| 10 | MMD | 53 | F | Aspirin 100 mg/day | Cefazolin 1g | TIA | 4 months | Bilateral | - | - | GSE157628 |
| 11 | MMD | 43 | F | Aspirin 100 mg/day,  telmisartan 80 mg/day | Cefazolin 1g | TIA | 10 months | Bilateral | - | - | GSE157628 |
| 12 | IA | 62 | F | No | Cefazolin 1g | No | - | - | Supraclinoid/  27mm | - | GSE157628 |
| 13 | IA | 78 | F | No | Cefazolin 1g | Oculomotor  nerve palsy | 5 months | - | Cavernous/30mm | - | GSE157628 |
| 14 | IA | 79 | F | Cilostazol 100 mg/day,  candesartan 8 mg/day,  benidipine 4 mg/day,  rosuvastatine 5 mg/day, ezetimibe 10 mg/day | Cefmetazole 1g | Oculomotor  nerve palsy | 6 months | - | Cavernous/25mm | - | GSE157628 |
| 15 | IA | 70 | F | Clopidogrel 75 mg/day, valsartan 160 mg/day, amlodipine 10 mg/day, imidapril 5 mg/day, doxazosin 2 mg/day, methotrexate  10 mg/week | Cefazolin 1g | Oculomotor  nerve palsy | 7 months | - | Cavernous/20mm | - | GSE157628 |
| 16 | IA | 65 | F | Clopidogrel 75 mg/day, atorvastatin  10 mg/day | Cefazolin 1g | No | - | - | Cavernous/26mm | - | GSE157628 |
| 17 | IA | 71 | F | Aspirin 100 mg/day,  valsartan 160 mg/day,  amlodipine 5 mg/day | Cefazolin 1g | No | - | - | Supraclinoid/22mm | - | GSE157628 |
| 18 | EPI | 56 | M | Pravastatin 20 mg,  levetiracetam 2000 mg/day, lamotrigine  300 mg/day,  perampanel 4 mg/day | Cefazolin 1g | Seizures | Several days | - | - | Temporal  lobe | GSE157628 |
| 19 | EPI | 14 | M | Lacosamide 300 mg/day, perampanel 6 mg/day, clobazam 15 mg/day | Cefazolin 1g | Seizures | Several days | - | - | Frontal lobe | GSE157628 |
| 20 | EPI | 20 | M | Lacosamide 300 mg/day, lamotrigine  200 mg/day,  carbamazepine 400 mg/day | Cefazolin 1g | Seizures | Several days | - | - | Frontal lobe | GSE157628 |
| 21 | MMD | 64 | F | Cilostazol 100 mg/day,  atorvastatin 10 mg/day | Cefazolin 1 g | ICH | 2 months | One side | - | - | GSE189993 |
| 22 | MMD | 47 | F | Aspirin 100 mg/day,  Lansoprazole 15 mg/day,  Atorvastatin Calcium  Hydrate 10 mg/day,  Tsumura Goreisan 2.5  g/day, Bepotastine  Besilate 10 mg/day | Cefazolin 1 g | IF | 8 months | Bilateral | - | - | GSE189993 |
| 23 | MMD | 48 | F | Amlodipine Besilate 5  mg/day | Cefazolin 1 g | ICH | 6 months | Bilateral | - | - | GSE189993 |
| 24 | MMD | 49 | F | Aspirin 100 mg/day | Cefazolin 1 g | TIA | 9 months | Bilateral | - | - | GSE189993 |
| 25 | MMD | 59 | F | Cilostazol 100 mg/day,  Esomeprazole Magnesium  Hydrate 20 mg/day | Cefazolin 1 g | IF | 11 months | Bilateral | - | - | GSE189993 |
| 26 | MMD | 50 | F | Cilostazol 100 mg/day,  Olopatadine  Hydrochloride 5 mg/day | Cefazolin 1 g | TIA | 1 month | Bilateral | - | - | GSE189993 |
| 27 | MMD | 45 | F | Aspirin 100 mg/day,  Lansoprazole 15 mg/day,  Clopidogrel Sulfate 75  mg/day, Pitavastatin Calcium Hydrate 2  mg/day | Cefazolin 1 g | IF | 1 year and  1 month | Bilateral | - | - | GSE189993 |
| 28 | MMD | 48 | F | Aspirin 100 mg/day,  Lansoprazole 15 mg/day,  Celecoxib 100 mg/day | Cefazolin 1 g | TIA | 3 months | Bilateral | - | - | GSE189993 |
| 29 | MMD | 51 | M | Aspirin 100 mg, Etizolam  0.5 mg/day, Olmesartan  Medoxomil 10 mg/day,  Azelnidipine 8 mg/day | Cefazolin 1 g | TIA | 1 month | Bilateral | - | - | GSE189993 |
| 30 | MMD | 53 | F | Aspirin 100 mg/day,  Lansoprazole 15 mg/day | Cefazolin 1 g | TIA | 7 months | Bilateral | - | - | GSE189993 |
| 31 | MMD | 43 | F | Aspirin 100 mg, /day  Lansoprazole 15 mg/day,  Telmisartan 40 mg/day | Cefazolin 1 g | TIA | 1 year and 2 months | Bilateral | - | - | GSE189993 |
| 32 | MMD | 34 | F | Aspirin 100 mg/day | Cefazolin 1 g | TIA | 5 months | Bilateral | - | - | GSE189993 |
| 33 | MMD | 29 | F | Levetiracetam 1000  mg/day, Phenytoin 300  mg/day, Sodium Ferrous  Citrate 100 mg/day | Cefazolin 1 g | ICH | 7 months | Bilateral | - | - | GSE189993 |
| 34 | MMD | 32 | M | Aspirin 100 mg/day,  Lansoprazole 15 mg/day | Cefazolin 1 g | TIA | Several days | Bilateral | - | - | GSE189993 |
| 35 | MMD | 7 | F | No | Cefazolin 1 g | TIA | Several days | Bilateral | - | - | GSE189993 |
| 36 | MMD | 27 | M | Aspirin 100 mg/day,  Esomeprazole Magnesium  Hydrate 20 mg/day | Cefazolin 1 g | TIA | 8 months | Bilateral | - | - | GSE189993 |
| 37 | MMD | 5 | M | Aspirin 30 mg/day | Cefazolin 0.3 g | IF | 3 months | Bilateral | - | - | GSE189993 |
| 38 | MMD | 39 | M | No | Cefazolin 1 g | TIA | 7 months | Bilateral | - | - | GSE189993 |
| 39 | MMD | 11 | M | Cilostazol 150 mg/day | Cefazolin 0.5 g | TIA | 2 months | Bilateral | - | - | GSE189993 |
| 40 | MMD | 46 | F | Sodium Ferrous Citrate 50  mg/day, Olopatadine  Hydrochloride 10 mg/day | Cefazolin 1 g | TIA | 2 months | Bilateral | - | - | GSE189993 |
| 41 | MMD | 35 | F | Pregabalin 225 mg/day,  Amitriptyline  Hydrochloride 35 mg/day,  Desloratadine 5 mg/day | Cefazolin 1 g | TIA | 8 months | Bilateral | - | - | GSE189993 |
| 42 | IA | 62 | F | Loxoprofen Sodium  Hydrate 180 mg/day,  Rebamipide 300 mg/day,  Brotizolam 0.25 mg/day,  Diazepam 2 mg/day | Cefazolin 1 g | No | - | - | ICA/27mm | - | GSE189993 |
| 43 | IA | 78 | F | Etizolam 0.5 mg/day,  Tandospirone Citrate 20  mg/day, Kallidinogenase 100 units/day, Magnesium  Oxide 660 mg/day | Cefazolin 1 g | No | - | - | Covernous/28mm | - | GSE189993 |
| 44 | IA | 79 | F | Aspirin 100 mg/day,  Cilostazol 100 mg/day,  Acetaminophen 1200  mg/day, Mecobalamin  1500 μg/day, Benidipine  Hydrochloride 4 mg/day,  Ezetimibe 10 mg/day,  Rosuvastatin Calcium 5  mg/day, Candesartan  Cilexetil 8 mg/day | Cefazolin 1 g | Oculomotor  nerve palsy | 1 month | - | Cavernous/26mm | - | GSE189993 |
| 45 | IA | 70 | F | Valsartan 160 mg/day,  Amlodipine Besilate 10  mg/day, Imidapril  Hydrochloride 5 mg/day,  Doxazosin Mesilate 2  mg/day, methotrexate 10  mg/week | Cefazolin 1 g | Oculomotor  nerve palsy | Several days | - | Cavernous/20mm | - | GSE189993 |
| 46 | IA | 65 | F | Clopidogrel Sulfate 75  mg/day, Atorvastatin  Calcium Hydrate 10  mg/day, L -Carbocisteine  1500 mg, Mecobalamin  1500 μg/day,  d -Chlorpheniramine  Maleate 6 mg/day,  Betamethasone 0.5  mg/day,  d -Chlorpheniramine  Maleate 4 mg/day,  Olopatadine  Hydrochloride 20 mg/day | Cefazolin 1 g | Oculomotor  nerve palsy,  head ache | Several days | - | Cavernous/27mm | - | GSE189993 |
| 47 | IA | 71 | F | Aspirin 100 mg/day,  Esomeprazole Magnesium  Hydrate 20 mg/day,  Valsartan 80 mg/day,  Amlodipine Besilate 5  mg/day, Suvorexant 15  mg/day | Cefazolin 1 g | No | - | - | ICA/21mm | - | GSE189993 |
| 48 | EPI | 56 | M | Levetiracetam 2000  mg/day, Lamotrigine 300  mg/day, Perampanel  Hydrate 4 mg/day,  Pravastatin Sodium 20  mg/day | Cefazolin 1 g | Seizures | Several days | - | - | Temporal  lobe | GSE189993 |
| 49 | EPI | 14 | M | Lacosamide 300 mg/day,  Clobazam 15 mg/day,  Perampanel Hydrate 6  mg/day | Cefazolin 1 g | Seizures | Several days | - | - | Frontal  lobe | GSE189993 |
| 50 | EPI | 20 | M | Lacosamide 400 mg/day,  Lamotrigine 200 mg/day,  Carbamazepine 400  mg/day | Cefazolin 1 g | Seizures | Several days | - | - | Frontal  lobe | GSE189993 |
| 51 | EPI | 43 | F | Carbamazepine 300  mg/day, Atorvastatin  Calcium Hydrate 5  mg/day, Fexofenadine  Hydrochloride 120  mg/day | Cefazolin 1 g | Seizures | Several days | - | - | Temporal  lobe | GSE189993 |
| 52 | EPI | 2 | M | Levetiracetam 325  mg/day, Zonisamid 16  mg/day | Cefotaxime 0.5 g | Seizures | Several days | - | - | Frontal  lobe | GSE189993 |
| 53 | MMD | 48 | F | Aspirin 100 mg/day, Lansoprazole 15 mg/day, Celecoxib 100 mg/day | Cefazolin 1 g | TIA | 3 months | Bilateral | - | - | GSE141024 |
| 54 | MMD | 51 | M | Aspirin 100 mg, Etizolam 0.5 mg/day, Olmesartan Medoxomil 10 mg/day, Azelnidipine 8 mg/day | Cefazolin 1 g | TIA | 1 months | Bilateral | - | - | GSE141024 |
| 55 | MMD | 53 | F | Aspirin 100 mg/day, Lansoprazole 15 mg/day | Cefazolin 1 g | TIA | 7 months | Bilateral | - | - | GSE141024 |
| 56 | MMD | 43 | F | Aspirin 100 mg, /day Lansoprazole 15 mg/day, Telmisartan 40 mg/day | Cefazolin 1 g | TIA | 14 months | Bilateral | - | - | GSE141024 |
| 57 | IA | 62 | F | Loxoprofen Sodium Hydrate 180 mg/day, Rebamipide 300 mg/day, Brotizolam 0.25 mg/day, Diazepam 2 mg/day | Cefazolin 1 g | None | - | - | ICA/27mm | - | GSE141024 |
| 58 | IA | 78 | F | Etizolam 0.5 mg/day, Tandospirone Citrate 20 mg/day, Kallidinogenase 100 units/day, Magnesium Oxide 660 mg/day | Cefazolin 1 g | None | - | - | Cavernous/28mm | - | GSE141024 |
| 59 | IA | 79 | F | Aspirin 100 mg/day, Cilostazol 100 mg/day, Acetaminophen 1200 mg/day, Mecobalamin 1500 μg/day, Benidipine Hydrochloride 4 mg/day, Ezetimibe 10 mg/day, Rosuvastatin Calcium 5 mg/day, Candesartan Cilexetil 8 mg/day | Cefazolin 1 g | Oculomotor  nerve palsy | 1 month | - | Cavernous/26mm | - | GSE141024 |
| 60 | IA | 70 | F | Valsartan 160 mg/day, Amlodipine Besilate 10 mg/day, Imidapril Hydrochloride 5 mg/day, Doxazosin Mesilate 2 mg/day, methotrexate 10 mg/week | Cefazolin 1 g | Oculomotor  nerve palsy | Several days | - | Cavernous/20mm | - | GSE141024 |

MMD, moyamoya disease; EPI, medically intractable seizures; IA, internal carotid artery aneurysm; M, male; F, female; ICH, intracerebral hemorrhage; IF, cerebral infarction; TIA, transient ischemic attack.

**Figure S1:** Analysis of immune infiltration and microenvironment for the four key genes.
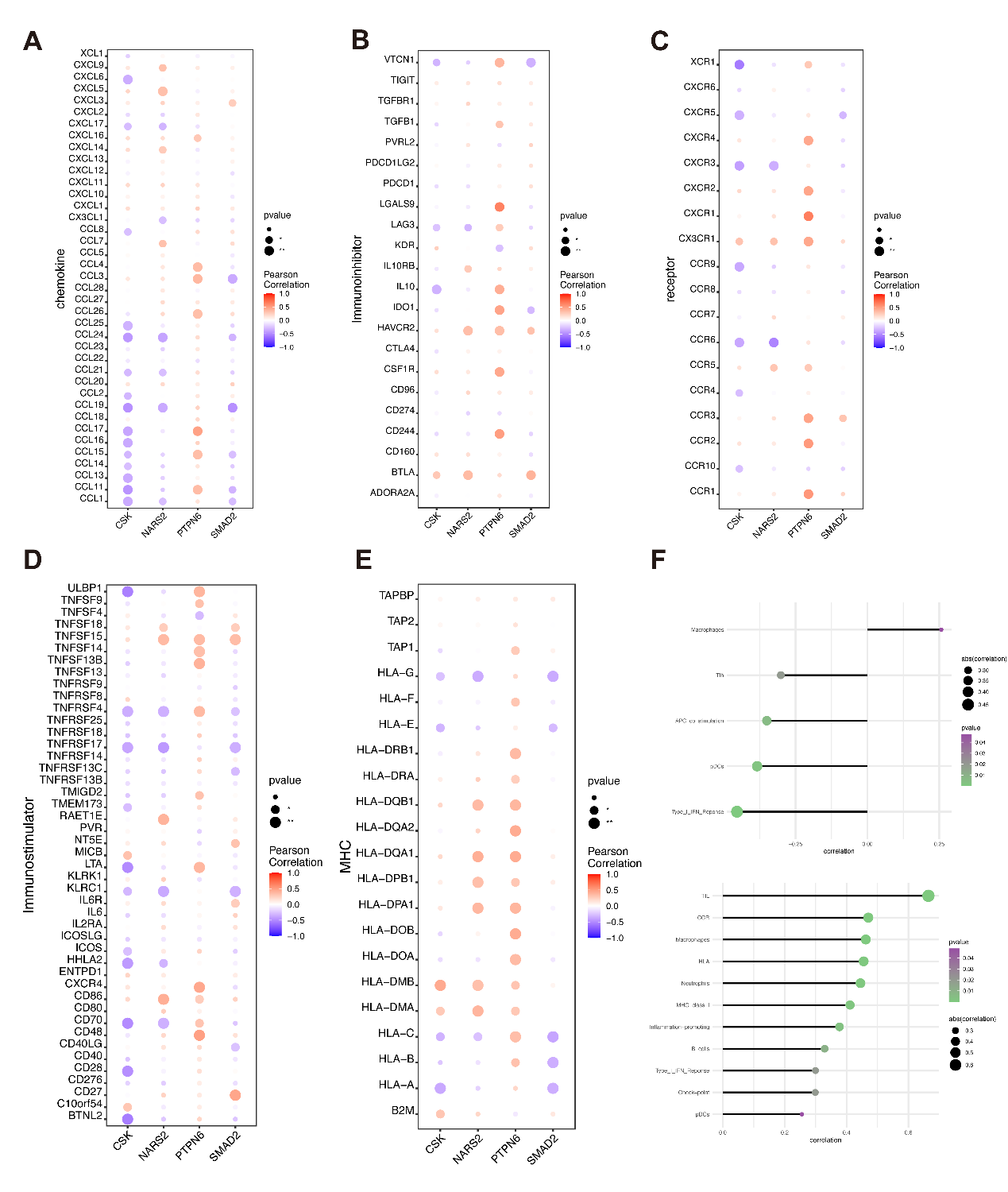
A, Bubble plot for the relationship between the 4 key genes and chemokine. B, Bubble plot for the relationship between the 4 key genes and immunoinhibitor. C, Bubble plot for the relationship between the 4 key genes and immunoinhibitory receptor. D, Bubble plot for the relationship between the 4 key genes and immunostimulatory. E, Bubble plot for the relationship between the 4 key genes and MHC. F, The relationship between the gene SMAD2 (upper of the figure) and PTPN6 (lower of the figure), and immune cells.
