# Supplementary material for: Identification of oxidative phosphorylation-related genes in moyamoya disease by combining bulk RNA-sequencing analysis and machine learning
Source: Front Genet. 2024 Jun 10;15:1417329. doi: 10.3389/fgene.2024.1417329 (PMC11197386; doi:10.3389/fgene.2024.1417329)
Supplement: Supplementary file 4 [file Table4.DOCX]

library(sva)

library(limma)

library(glmnet)

library(Boruta)

rt1=read.table("GSE141024.txt",sep="\t",header=T,check.names=F)

rt1=as.matrix(rt1)

rownames(rt1)=rt1[,1]

exp1=rt1[,2:ncol(rt1)]

dimnames1=list(rownames(exp1),colnames(exp1))

data1=matrix(as.numeric(as.matrix(exp1)),nrow=nrow(exp1),dimnames=dimnames1)

data1=avereps(data1)

rt2=read.table("GSE157628.txt",sep="\t",header=T,check.names=F)

rt2=as.matrix(rt2)

rownames(rt2)=rt2[,1]

exp2=rt2[,2:ncol(rt2)]

dimnames2=list(rownames(exp2),colnames(exp2))

data2=matrix(as.numeric(as.matrix(exp2)),nrow=nrow(exp2),dimnames=dimnames2)

data2=avereps(data2)

rt3=read.table("GSE189993.txt",sep="\t",header=T,check.names=F)

rt3=as.matrix(rt3)

rownames(rt3)=rt3[,1]

exp3=rt3[,2:ncol(rt3)]

dimnames3=list(rownames(exp3),colnames(exp3))

data3=matrix(as.numeric(as.matrix(exp3)),nrow=nrow(exp3),dimnames=dimnames3)

data3=avereps(data3)

sameGene=intersect(rownames(data1),rownames(data2))

sameGene=intersect(sameGene,rownames(data3))

data=cbind.data.frame(data1[sameGene,],data2[sameGene,],data3[sameGene,]) #,data4[sameGene,])

group1 <- read.table("Group-GSE141024.txt",sep = "\t",row.names = 1,check.names = F,stringsAsFactors = F,header = T)

group2 <- read.table("Group-GSE157628.txt",sep = "\t",row.names = 1,check.names = F,stringsAsFactors = F,header = T)

group3 <- read.table("Group-GSE189993.txt",sep = "\t",row.names = 1,check.names = F,stringsAsFactors = F,header = T)

tum.sam1 <- rownames(group1[which(group1$Group == 1),,drop = F])

tum.sam2 <- rownames(group2[which(group2$Group == 1),,drop = F])

tum.sam3 <- rownames(group3[which(group3$Group == 1),,drop = F])

nor.sam1 <- rownames(group1[which(group1$Group == 0),,drop = F])

nor.sam2 <- rownames(group2[which(group2$Group == 0),,drop = F])

nor.sam3 <- rownames(group3[which(group3$Group == 0),,drop = F])

tum.sam <- c(tum.sam1, tum.sam2, tum.sam3)# tum.sam4)

nor.sam <- c(nor.sam1, nor.sam2, nor.sam3)# nor.sam4)

data <- data[,c(tum.sam1,nor.sam1,tum.sam2,nor.sam2,tum.sam3,nor.sam3)]

write.table(data,file="merge.txt",sep="\t",quote=F,row.names = T,col.names = NA)

rt=read.table("merge.txt",sep="\t",header=T,check.names=F, row.names = 1,stringsAsFactors = F) ##??ȡ?ϲ????ݼ?

rt=as.matrix(rt)

batch <- data.frame(batch = rep(c("Group1","Group2","Group3"), times = c(nrow(group1),nrow(group2),nrow(group3))))

modcombat = model.matrix(~1, data=batch)

outTab <- as.data.frame(ComBat(dat=as.matrix(data), batch=batch$batch, mod=modcombat)) # change

outTab <- outTab[,c(nor.sam, tum.sam)]

write.table(outTab,file="normalize.txt",sep="\t",quote=F,row.names = T,col.names = NA)

library("FactoMineR")

library("factoextra")

pca.plot = function(data,col){

df.pca <- PCA(t(data), graph = FALSE)

fviz_pca_ind(df.pca,

geom.ind = "point",

col.ind = col ,

addEllipses = TRUE,

legend.title = "Groups"

)

}

data<-read.table("merge.txt",sep = "\t",row.names = 1,header = T)

group<-read.table("group.txt",sep = "\t",row.names = 1,header = T)

a<-pca.plot(data,factor(group$Group))

pdf(file="PCA-raw.pdf",width=6,height=5)

a

dev.off()

data1<-read.table("normalize.txt",sep = "\t",row.names = 1,header = T)

b<-pca.plot(data1,factor(group$Group))

pdf(file="PCA-after.pdf",width=6,height=5)

b

dev.off()

logFoldChange=1

adjustP=0.05

rt=read.table("normalize.txt",sep="\t",header=T,check.names=F)

rt=as.matrix(rt)

rownames(rt)=rt[,1]

exp=rt[,2:ncol(rt)]

dimnames=list(rownames(exp),colnames(exp))

rt=matrix(as.numeric(as.matrix(exp)),nrow=nrow(exp),dimnames=dimnames)

rt=avereps(rt)

modType=c(rep("con",length(nor.sam)),rep("treat",length(tum.sam)))

design <- model.matrix(~0+factor(modType))

colnames(design) <- c("con","treat")

fit <- lmFit(rt,design)

cont.matrix<-makeContrasts(treat-con,levels=design)

fit2 <- contrasts.fit(fit, cont.matrix)

fit2 <- eBayes(fit2)

allDiff=topTable(fit2,adjust='fdr',number=200000)

write.table(allDiff,file="limmaTab.xls",sep="\t",quote=F,row.names = T,col.names = NA)

diffSig <- allDiff[with(allDiff, (abs(logFC)>logFoldChange & P.Value < adjustP )), ]

write.table(diffSig,file="diff.xls",sep="\t",quote=F,row.names = T,col.names = NA)

diffUp <- allDiff[with(allDiff, (logFC>logFoldChange & P.Value < adjustP )), ]

write.table(diffUp,file="up.xls",sep="\t",quote=F,row.names = T,col.names = NA)

diffDown <- allDiff[with(allDiff, (logFC<(-logFoldChange) & P.Value < adjustP )), ]

write.table(diffDown,file="down.xls",sep="\t",quote=F,row.names = T,col.names = NA)

hmExp=rt[rownames(diffSig),]

diffExp=hmExp

write.table(diffExp,file="heatmap.txt",sep="\t",quote=F,col.names=NA,row.names = T)

pdf("1.Volcano.pdf")

xMax=max(abs(allDiff$logFC))

yMax=max(-log10(allDiff$P.Value))

plot(allDiff$logFC, -log10(allDiff$P.Value), xlab="logFC",ylab="-log10(P.Value)",

main="Volcano",xlim=c(-xMax,xMax),ylim=c(0,yMax),yaxs="i",pch=20, cex=0.8)

diffSub=subset(allDiff, P.Value<adjustP & logFC>logFoldChange)

points(diffSub$logFC, -log10(diffSub$P.Value), pch=20, col="palevioletred1",cex=0.8)

diffSub=subset(allDiff, P.Value<adjustP & logFC<(-logFoldChange))

points(diffSub$logFC, -log10(diffSub$P.Value), pch=20, col="dodgerblue",cex=0.8)

abline(v=0,lty=2,lwd=3)

dev.off()

library(limma)

library(pheatmap)

library(gplots)

plotdata=read.table("heatmap.txt",sep="\t",header=T,check.names=F,row.names = 1)

up=read.table("up.xls",header=T,stringsAsFactors = F,sep = "\t")

down=read.table("down.xls",header=T,stringsAsFactors = F,sep = "\t")

annCol <- data.frame(Group = rep(c("Control","Disease"),c(24,36)),

row.names = colnames(plotdata),

stringsAsFactors = F)

annRow <- data.frame(Direct = rep(c("Up","Down"),c(length(up$X),length(down$X))),

row.names = c(up$X,down$X),

stringsAsFactors = F)

annColors <- list("Group"=c("Control"="blue",

"Disease"="red"),

"Direct"=c("Up"="yellow",

"Down"="green"))

plotdata <- plotdata[c(up$X,down$X),]

plotdata <- t(scale(t(plotdata)))

plotdata[plotdata > 5] <- 5

plotdata[plotdata < -5] <- -5

pdf(file="heatmap.pdf",width = 8,height = 8)

pheatmap(plotdata,

scale = "none",

annotation_row=annRow,

annotation_col=annCol,

annotation_colors = annColors,

color = colorRampPalette(c("navy", "white", "palevioletred1"))(20),

#color = greenred(64),

fontsize_row=7,

fontsize_col=5,

fontsize=9,

cluster_cols = FALSE,

cluster_rows = FALSE,

show_colnames = F,

show_rownames = F)

dev.off()

library("org.Hs.eg.db")

rt=read.table("gene.txt",sep="\t",check.names=F,header=T)

genes=as.vector(rt[,1])

entrezIDs <- mget(genes, org.Hs.egSYMBOL2EG, ifnotfound=NA)

entrezIDs <- as.character(entrezIDs)

out=cbind(rt,entrezID=entrezIDs)

write.table(out,file="id.txt",sep="\t",quote=F,row.names=F)

library("clusterProfiler")

library("org.Hs.eg.db")

library("enrichplot")

library("ggplot2")

rt=read.table("id.txt",sep="\t",header=T,check.names=F)

rt=rt[is.na(rt[,"entrezID"])==F,]

gene=rt$entrezID

kk <- enrichGO(gene = gene,

OrgDb = org.Hs.eg.db,

pvalueCutoff =0.05,

qvalueCutoff = 0.05,

ont="all",

readable =T)

write.table(kk,file="GO.txt",sep="\t",quote=F,row.names = F)

pdf(file="GObarplot.pdf",width = 10,height = 13)

barplot(kk, drop = TRUE, showCategory =10,split="ONTOLOGY",label_format=100) + facet_grid(ONTOLOGY~., scale='free')

dev.off()

pdf(file="GObubble.pdf",width = 10,height = 15)

dotplot(kk,showCategory = 10,split="ONTOLOGY") + facet_grid(ONTOLOGY~., scale='free')

dev.off()

library("clusterProfiler")

library("org.Hs.eg.db")

library("enrichplot")

library("ggplot2")

rt=read.table("id.txt",sep="\t",header=T,check.names=F)

rt=rt[is.na(rt[,"entrezID"])==F,]

gene=rt$entrezID

kk <- enrichKEGG(gene = gene, organism = "hsa", pvalueCutoff =0.05, qvalueCutoff =0.05)

write.table(kk,file="KEGGId.txt",sep="\t",quote=F,row.names = F)

pdf(file="KEGGbarplot.pdf",width = 10,height = 10)

barplot(kk, drop = TRUE, showCategory = 30,label_format=100)

dev.off()

pdf(file="KEGGbubble.pdf",width = 10,height = 10)

dotplot(kk, showCategory = 30)

dev.off()

library(tidyverse)

library(glmnet)

source('msvmRFE.R')

library(VennDiagram)

library(sigFeature)

library(e1071)

library(caret)

library(randomForest)

train<-read.table("ARGexp.txt",row.names = 1,as.is = F,header = T)

train[1:4,1:4]

x <- as.matrix(train[,-1])

(y <- ifelse(train$group == "NR", 0,1))

x[1:4,1:4]

set.seed(10)

fit = glmnet(x, y, family = "gaussian", alpha = 1, lambda = NULL)

plot(fit, xvar = "dev", label = TRUE)

cvfit = cv.glmnet(x, y,

nfold=10,

family = "gaussian", type.measure = "class")

plot(cvfit)

cvfit$lambda.min

myCoefs <- coef(cvfit, s="lambda.min");

lasso_fea <- myCoefs@Dimnames[[1]][which(myCoefs != 0 )]

(lasso_fea <- lasso_fea[-1])

write.csv(lasso_fea,"feature_lasso.csv")

predict <- predict(cvfit, newx = x[1:nrow(x),], s = "lambda.min", type = "class")

table(predict,y)

input <- train

set.seed(10)

svmRFE(input, k = 5, halve.above = 100)

nfold = 5

nrows = nrow(input)

folds = rep(1:nfold, len=nrows)[sample(nrows)]

folds = lapply(1:nfold, function(x) which(folds == x))

results = lapply(folds, svmRFE.wrap, input, k=5, halve.above=100)

top.features = WriteFeatures(results, input, save=F)

head(top.features)

write.csv(top.features,"feature_svm.csv")

featsweep = lapply(1:10, FeatSweep.wrap, results, input)

featsweep

no.info = min(prop.table(table(input[,1])))

errors = sapply(featsweep, function(x) ifelse(is.null(x), NA, x$error))

PlotErrors(errors, no.info=no.info)

Plotaccuracy(1-errors,no.info=no.info)

which.min(errors)

(myoverlap <- intersect(lasso_fea, top.features[1:which.min(errors), "FeatureName"]))

summary(lasso_fea%in%top.features[1:which.min(errors), "FeatureName"])

pdf("C_lasso_SVM_venn.pdf", width = 5, height = 3)

grid.newpage()

venn.plot <- venn.diagram(list(LASSO = lasso_fea,

SVM_RFE = as.character(top.features[1:which.min(errors),"FeatureName"])), NULL,

fill = c("#E31A1C","#E7B800"),

alpha = c(0.5,0.5), cex = 4, cat.fontface=3,

category.names = c("LASSO", "SVM_RFE"),

main = "Overlap")

grid.draw(venn.plot)

dev.off()

inputFile="normalize.txt"

gmtFile="immune.gmt"

library(GSVA)

library(limma)

library(GSEABase)

rt=read.table(inputFile,sep="\t",header=T,check.names=F)

rt=as.matrix(rt)

rownames(rt)=rt[,1]

exp=rt[,2:ncol(rt)]

dimnames=list(rownames(exp),colnames(exp))

mat=matrix(as.numeric(as.matrix(exp)),nrow=nrow(exp),dimnames=dimnames)

mat=avereps(mat)

mat=mat[rowMeans(mat)>0,]

geneSet=getGmt(gmtFile,

geneIdType=SymbolIdentifier())

ssgseaScore=gsva(mat, geneSet, method='ssgsea', kcdf='Gaussian', abs.ranking=TRUE)

normalize=function(x){

return((x-min(x))/(max(x)-min(x)))}

ssgseaOut=normalize(ssgseaScore)

ssgseaOut=rbind(id=colnames(ssgseaOut),ssgseaOut)

write.table(ssgseaOut,file="ssgseaOut.txt",sep="\t",quote=F,col.names=F)

library(RcisTarget)

library(visNetwork)

library(reshape2)

library(stringr)

library(ggplot2)

library(ggpubr)

library(AUCell)

library(limma)

library(e1071)

library(Hmisc)

library(dplyr)

library(aplot)

library(doRNG)

library(doMC)

library(DT)

GseGroup <- read.table("group.txt",header=T,sep="\t",check.names=F)

compare_method = "anova"

ciberRes=read.table("ssgseaOut.txt",sep="\t",header=T,row.names=1,check.names=F)

ciber <- ciberRes[,setdiff(colnames(ciberRes), c("P-value", "Correlation", "RMSE"))]

ciber$Acc <- rownames(ciber)

if(sort(unique(GseGroup$Tissue))[1] == "Control"){

datGroup <- GseGroup[order(GseGroup$Tissue),]

}else{

datGroup <- GseGroup[order(GseGroup$Tissue, decreasing = T),]

}

datGroup$Acc <- factor(datGroup$Acc, levels = datGroup$Acc)

data <- dplyr::inner_join(datGroup,ciber,by="Acc")

data_p <- melt(data, id.vars = colnames(datGroup))

head(data_p)

data_p$Acc <- factor(data_p$Acc, levels = datGroup$Acc)

datGroup$p <- "Group"

p2 <- ggplot(datGroup,aes(Acc, p, fill=Tissue)) +

geom_tile() +

scale_fill_manual(values=c("#1CFA04", "#C705FF")) +

scale_y_discrete(position = "right") +

theme_minimal()+xlab(NULL) + ylab(NULL) +

theme(text = element_text(size = 15)) +

theme(axis.text.x = element_blank())+

labs(fill = "Group")

sample_names <- datGroup$Acc

p1 <- ggplot(data_p, aes(x = Acc, y = value, fill=variable)) +

geom_bar(stat="identity", position = "fill", width = 0.5) +

geom_col(position = 'fill', width = 0.6) +

guides(fill=guide_legend(title = NULL)) +

ylab("Relative Percent") + xlab("") +

theme_bw() +

theme(axis.ticks.length=unit(0.5,'cm')) +

theme(panel.grid.major = element_blank(), panel.grid.minor = element_blank()) +

theme(axis.text=element_text(size = 15)) +

scale_x_discrete(labels = sample_names) +

theme(axis.text.y=element_text(colour = "black", vjust=0,size = 15)) +

theme(axis.title =element_text(size = 20)) +

theme(text = element_text(size = 15)) +

scale_y_continuous(expand=c(0,0.05))+

theme(axis.text.x = element_text(angle = 45, hjust = 1,size = 10))

p <- p1 %>% insert_top(p2, height = 0.05)

ggsave(p, filename = "Immune infiltration.pdf", width = (nrow(datGroup)/3)+3, height = 8)

rt=read.table("ssgseaOut.txt",sep="\t",header=T,row.names=1,check.names=F)

library(corrplot)

pdf("corHeatmap.pdf",height=13,width=13)

corrplot(corr=cor(rt),

method = "color",

order = "hclust",

tl.col="black",

addCoef.col = "black",

number.cex = 1,

col=colorRampPalette(c("blue", "white", "red"))(50),

)

dev.off()

library(vioplot)

normal=24

tumor=36

rt=read.table("ssgseaOut.txt",sep="\t",header=T,row.names=1,check.names=F)

pdf("vioplot.pdf",height=8,width=15)

par(las=1,mar=c(10,6,3,3))

x=c(1:ncol(rt))

y=c(1:ncol(rt))

plot(x,y,

xlim=c(0,55),ylim=c(min(rt),max(rt)+0.02),

main="",xlab="", ylab="Fraction",

pch=21,

col="white",

xaxt="n")

text(seq(1,55,3),-0.05,xpd = NA,labels=colnames(rt),cex = 1,srt = 45,pos=2)

for(i in 1:ncol(rt)){

normalData=rt[1:normal,i]

tumorData=rt[(normal+1):(normal+tumor),i]

vioplot(normalData,at=3*(i-1),lty=1,add = T,col = 'blue')

vioplot(tumorData,at=3*(i-1)+1,lty=1,add = T,col = 'red')

wilcoxTest=wilcox.test(normalData,tumorData)

p=round(wilcoxTest$p.value,3)

mx=max(c(normalData,tumorData))

lines(c(x=3*(i-1)+0.2,x=3*(i-1)+0.8),c(mx,mx))

text(x=3*(i-1)+0.5,y=mx+0.02,labels=ifelse(p<0.001,paste0("p<0.001"),paste0("p=",p)),cex = 0.8)

}

dev.off()

library(ggplot2)

library(ggpubr)

library(SimDesign)

library(cowplot)

library(dplyr)

library(GSVA)

library(limma)

library(stringr)

jco <- c("#2874C5","#EABF00","#868686","#C6524A","#80A7DE")

expr <- read.table("normalize.txt",sep = "\t",row.names = 1,check.names = F,stringsAsFactors = F,header = T)

gene <- read.table("genelist.txt",sep = "\t",row.names = NULL,check.names = F,stringsAsFactors = F,header = F)

ciber <- read.table("ssgseaOut.txt",sep = "\t",row.names = 1,check.names = F,stringsAsFactors = F,header = T)

for (i in gene$V1) {

message(paste0("analysis of ",i," starts..."))

subexpr <- as.numeric(expr[i,])

names(subexpr) <- colnames(expr)

lsam <- names(subexpr[subexpr < median(subexpr)])

hsam <- names(subexpr[subexpr >= median(subexpr)])

dat <- as.numeric(expr[i,]); names(dat) <- colnames(expr)

comsam <- intersect(names(dat), rownames(ciber))

tmp1 <- dat[comsam]

tmp2 <- ciber[comsam,]

var <- colnames(ciber)

data <- data.frame(var)

for (j in 1:length(var)){

test <- cor.test(as.numeric(tmp2[,j]),tmp1,method = "pearson")

data[j,2] <- test$estimate

data[j,3] <- test$p.value

}

names(data) <- c("symbol","correlation","pvalue")

data <- as.data.frame(na.omit(data))

data %>%

filter(pvalue <0.05) %>%

ggplot(aes(correlation,forcats::fct_reorder(symbol,correlation))) +

geom_segment(aes(xend=0,yend=symbol)) +

geom_point(aes(col=pvalue,size=abs(correlation))) +

scale_colour_gradientn(colours=c("#7fc97f","#984ea3")) +

scale_size_continuous(range =c(2,8)) +

theme_minimal() +

ylab(NULL)

ggsave(paste0("correlation between cibersort and expression of ", i,".pdf"),width = 8,height = 6)

}

library(stringr)

a_1 <- read.table("normalize.txt",header = T,row.names = 1,sep = "\t", quote = "",fill = T,check.names=F)

dim(a_1)

head(a_1[,1:3])

a_2 <- as.data.frame(t(a_1))

dim(a_2)

head(a_2[,1:3])

a_3 <- a_1

a_3$Id <- rownames(a_3)

dim(a_3)

head(a_3[,1:3])

b_1 <- read.table("Immunomodulator_and_chemokines.txt",header = T,sep = "\t", quote = "",fill = T)

dim(b_1)

head(b_1)

b_2 <- b_1[b_1$type == "chemokine",]

dim(b_2)

head(b_2)

data1 <- dplyr::inner_join(b_2,a_3,by="Id")

dim(data1)

head(data1[,1:6])

data2 <- a_2[,c("CSK","NARS2","PTPN6","SMAD2",data1$Id)]

dim(data2)

head(data2[,1:5])

library(Hmisc)

CorMatrix <- function(cor,p) {

ut <- upper.tri(cor)

data.frame(row = rownames(cor)[row(cor)[ut]] ,

column = rownames(cor)[col(cor)[ut]],

cor =(cor)[ut],

p = p[ut] )

}

res <- rcorr(as.matrix(data2),type = "spearman")

result_1 <- CorMatrix(res$r, res$P)

head(result_1)

dim(result_1)

result_2 <- result_1[result_1$row == "CSK" |result_1$row == "NARS2"|result_1$row == "PTPN6"|result_1$row == "SMAD2",]

dim(result_2)

head(b_2)

b_2$column <- b_2$Id

head(b_2)

result_3 <- dplyr::inner_join(result_2,b_2,by="column")

dim(result_3)

result1 <- result_3[,1:4]

head(result1)

dim(result1)

result1$Regulation <- result1$cor

result1[,5][result1[,5] > 0] <- c("postive")

result1[,5][result1[,5] < 0] <- c("negative")

head(result1)

colnames(result1) <- c("gene", "immuneGene", "cor", "pvalue", "Regulation")

write.table(result1,file="chemokine.xls",sep="\t",quote=F,col.names=T,row.names = F)

a1 <- read.table("chemokine.xls",header = T,sep = "\t", quote = "",fill = T)

head(a1)

data2 <- a1

library(ggpubr)

data2$pvalue <- ifelse(data2$pvalue < 0.05,

ifelse(data2$pvalue < 0.01,"**","*"),

"")

data2$pvalue[1:20]

data2$type <- data2$cor

summary(data2)

data3 <- data2[order(data2$immuneGene,data2$cor),]

head(data3)

dim(data3)

data4 <- data3[data3$pvalue < 0.05,]

dim(data4)

summary(data4)

p <- ggplot(data4,aes(x=gene,y=immuneGene)) +

geom_point(aes(colour = cor, size=pvalue)) +

labs(x="",y="chemokine")

p <- p + scale_colour_gradient2(low = "blue", high = "red", mid = "white",

midpoint = 0, limit = c(-1, 1), space = "Lab",

name="Pearson\nCorrelation")

p <- p + theme_bw() +

theme(panel.grid.major = element_blank(), panel.grid.minor = element_blank()) +

theme(axis.text=element_text(size = 15)) +

theme(axis.text.x=element_text(colour = "black",angle=0,hjust=0.5,size = 15)) +

theme(axis.text.y=element_text(colour = "black", vjust=0,size = 15)) +

theme(axis.title =element_text(size = 20)) +

theme(text = element_text(size = 15))

p+rotate_x_text(45)

ggsave("chemokine.pdf")

library(AUCell)

library(RcisTarget)

library(doMC)

library(doRNG)

library(DT)

library(visNetwork)

data("motifAnnotations_hgnc_v9")

gene <- read.table("keygene.txt",sep = "\t",row.names = 1,check.names = F,stringsAsFactors = F,header = T)

geneList1 <- rownames(gene)

head(geneList1)

geneLists <- list(key_gene=geneList1)

data(motifAnnotations_hgnc)

library(RcisTarget.hg19.motifDBs.cisbpOnly.500bp)

data(hg19_500bpUpstream_motifRanking_cispbOnly)

motifRankings <- hg19_500bpUpstream_motifRanking_cispbOnly

motifRankings

motifEnrichmentTable_wGenes <- cisTarget(geneLists, motifRankings,

motifAnnot=motifAnnotations_hgnc_v9)

head(motifEnrichmentTable_wGenes)

motifEnrichmentTable_wGenes_wLogo <- addLogo(motifEnrichmentTable_wGenes)

resultsSubset <- motifEnrichmentTable_wGenes_wLogo[1:10,]

datatable(resultsSubset[,-c("rankAtMax","TF_lowConf"), with=FALSE],

escape = FALSE,

filter="top", options=list(pageLength=5))

write.table(data.frame('ID'=row.names(motifEnrichmentTable_wGenes),motifEnrichmentTable_wGenes),file='onco_matrix1.txt',sep='\t',quote=F,row.names = F)

motifs_AUC <- calcAUC(geneLists, motifRankings, nCores=1)

(motifs_AUC )

par(mfrow = c(1,2))

pdf(file="motif enrichment.pdf",width=8,height=8)

for(i in names(geneLists)){

auc <- getAUC(motifs_AUC)[i,]

hist(auc, main=i, xlab="AUC histogram",

breaks=100, col="#ff000050", border="darkred")

nes3 <- (3*sd(auc)) + mean(auc)

abline(v=nes3, col="red")

}

dev.off()

motifEnrichmentTable <- addMotifAnnotation(motifs_AUC, nesThreshold=3,

motifAnnot=motifAnnotations_hgnc_v9,

highlightTFs=list(lasso_gene="CEBPB"))

head(motifEnrichmentTable[,-"TF_lowConf", with=FALSE])

motifEnrichmentTable_wGenes <- addSignificantGenes(motifEnrichmentTable,

rankings=motifRankings,

geneSets=geneLists)

motifEnrichmentTable_wGenes[1:4,]

geneSetName <- names(geneLists)[1]

selectedMotifs <- c("cisbp__M5965","cisbp__M6113","cisbp__M5321")

pdf(file="motif enrichment best.pdf",width=8,height=8)

par(mfrow=c(2,2))

getSignificantGenes(geneLists[[geneSetName]],

motifRankings,

signifRankingNames=selectedMotifs,

plotCurve=TRUE, maxRank=5000, genesFormat="none",

method="aprox")

dev.off()

library(ggpubr)

pFilter=0.99

rt=read.table("ARGexp.txt",sep="\t",header=T,row.names=1,check.names=F)

data=rt

Type=read.table("cluster.Immunity.txt",sep="\t",check.names=F,row.names=1,header=F)

Type=Type[row.names(data),]

colnames(Type)=c("cluster","Subtype")

outTab=data.frame()

data=cbind(data,Type)

for(i in colnames(data[,1:(ncol(data)-2)])){

rt1=data[,c(i,"Subtype")]

colnames(rt1)=c("expression","Subtype")

ksTest<-kruskal.test(expression ~ Subtype, data = rt1)

pValue=ksTest$p.value

if(pValue<pFilter){

outTab=rbind(outTab,cbind(rt1,gene=i))

print(pValue)

}

}

write.table(outTab,file="data.txt",sep="\t",row.names=F,quote=F)

data=read.table("data.txt",sep="\t",header=T,check.names=F)

data$Subtype=factor(data$Subtype, levels=c("Control","Disease"))

p=ggboxplot(data, x="gene", y="expression",color = "grey",fill = "Subtype",

ylab="Expression",

xlab="",

palette =c("skyblue","pink") )

p=p+rotate_x_text(45)

p

pdf(file="boxplot.pdf",width=12,height=4)

p+stat_compare_means(aes(group=Subtype),symnum.args=list(cutpoints = c(0, 0.001, 0.01, 0.05, 1), symbols = c("***", "**", "*", "ns")),label = "p.signif",method="t.test")

dev.off()

library(dplyr)

library(ggplot2)

data %>%

filter(Subtype %in% c("Control","Disease")) %>%

ggplot(aes(x= gene, y= expression, fill = Subtype, color = Subtype))+

geom_boxplot(alpha=0.3)+

scale_fill_manual(name= "Subtype", values = c("deepskyblue", "hotpink"))+

scale_color_manual(name = "Subtype", values = c("dodgerblue", "plum3"))+

theme_bw()+labs(x="", y="Expression")+

theme(axis.text.x = element_text( vjust = 1,size = 12, hjust = 1,colour = "black"),legend.position="top")+

rotate_x_text(45)+stat_compare_means(aes(group=Subtype),symnum.args=list(cutpoints = c(0, 0.001, 0.01, 0.05, 1), symbols = c("***", "**", "*", "ns")),label = "p.signif",method="t.test")

library(ggplot2)

library(stringr)

a_1 <- read.table("symbol.txt",header = T,row.names = 1,sep = "\t", quote = "",fill = T,check.names=F)

dim(a_1)

head(a_1[,1:3])

a_2 <- as.data.frame(t(a_1))

dim(a_2)

head(a_2[,1:3])

a_3 <- a_1

a_3$Id <- rownames(a_3)

dim(a_3)

head(a_3[,1:3])

b_1 <- read.table("111.txt",header = T,sep = "\t", quote = "",fill = T)

dim(b_1)

head(b_1)

b_2 <- b_1[b_1$type == "Disease",]

dim(b_2)

head(b_2)

data1 <- dplyr::inner_join(b_2,a_3,by="Id")

dim(data1)

head(data1[,1:6])

data2 <- a_2[,c("CSK", "NARS2","PTPN6","SMAD2",data1$Id)]

dim(data2)

head(data2[,1:5])

library(Hmisc)

CorMatrix <- function(cor,p) {

ut <- upper.tri(cor)

data.frame(row = rownames(cor)[row(cor)[ut]] ,

column = rownames(cor)[col(cor)[ut]],

cor =(cor)[ut],

p = p[ut] )

}

res <- rcorr(as.matrix(data2),type = "pearson")

result_1 <- CorMatrix(res$r, res$P)

head(result_1)

dim(result_1)

result_2 <- result_1[result_1$row == "CSK" |result_1$row == "NARS2" |result_1$row == "PTPN6"|result_1$row == "SMAD2",]

dim(result_2)

head(b_2)

b_2$column <- b_2$Id

head(b_2)

result_3 <- dplyr::inner_join(result_2,b_2,by="column")

dim(result_3)

result1 <- result_3[,1:4]

head(result1)

dim(result1)

result1$Regulation <- result1$cor

result1[,5][result1[,5] > 0] <- c("postive")

result1[,5][result1[,5] < 0] <- c("negative")

head(result1)

colnames(result1) <- c("gene", "immuneGene", "cor", "pvalue", "Regulation")

write.table(result1,file="MMD.xls",sep="\t",quote=F,col.names=T,row.names = F)

a1 <- read.table("MMD.xls",header = T,sep = "\t", quote = "",fill = T)

head(a1)

data2 <- a1

library(ggpubr)

data2$pvalue <- ifelse(data2$pvalue < 0.05,

ifelse(data2$pvalue < 0.01,"**","*"),

"")

data2$pvalue[1:20]

data2$type <- data2$cor

summary(data2)

data3 <- data2[order(data2$immuneGene,data2$cor),]

head(data3)

dim(data3)

data4 <- data3[data3$pvalue < 0.05,]

dim(data4)

summary(data4)

p <- ggplot(data4,aes(x=gene,y=immuneGene)) +

geom_point(aes(colour = cor, size=pvalue)) +

labs(x="",y="MMD genes")

p <- p + scale_colour_gradient2(low = "blue", high = "red", mid = "white",

midpoint = 0, limit = c(-1, 1), space = "Lab",

name="Pearson\nCorrelation")

p <- p + theme_bw() +

theme(panel.grid.major = element_blank(), panel.grid.minor = element_blank()) +

theme(axis.text=element_text(size = 15)) +

theme(axis.text.x=element_text(colour = "black",angle=0,hjust=0.5,size = 15)) +

theme(axis.text.y=element_text(colour = "black", vjust=0,size = 15)) +

theme(axis.title =element_text(size = 20)) +

theme(text = element_text(size = 15))

p+rotate_x_text(45)

ggsave("MMD.pdf")

library(ggplot2)

library(ggExtra)

rt=read.table("symbol.txt",sep="\t",header=T,check.names=F,row.names = 1)

dat<-as.data.frame(t(rt))

corr_eqn <- function(x,y,digits=3) {

test <- cor.test(x,y,type="pearson")

paste(paste0("n = ",length(x)),

paste0("r = ",round(test$estimate,digits),"(Pearson)"),

paste0("p.value= ",round(test$p.value,digits)),

sep = ", ")

}

gene<-as.numeric(dat$CSK)

imucell<-dat$APOE

corr_eqn(gene,imucell)

gg<-ggplot(dat, aes(x=gene, y=imucell)) +

geom_point(color = "black") +

geom_smooth(method="loess", se=F,color="blue") +

labs(

y="APOE",

x="CSK",

title="Scatterplot")+

labs(title = paste0(corr_eqn(gene,imucell)))+

theme_bw()

gg

gg2 <- ggMarginal(gg, type="density")

gg2 <- ggMarginal(gg, type="density",xparams = list(fill ="orange"),

yparams = list(fill ="skyblue"))
